# Why Is a High Temperature Needed by *Thermus thermophilus* Argonaute During mRNA Silencing: A Theoretical Study

**DOI:** 10.3389/fchem.2018.00223

**Published:** 2018-06-14

**Authors:** Ye Liu, Zhengfei Yu, Jingxuan Zhu, Song Wang, Dong Xu, Weiwei Han

**Affiliations:** ^1^Key Laboratory for Molecular Enzymology and Engineering of Ministry of Education, School of Life Science, Jilin University, Changchun, China; ^2^State Key Laboratory of Theoretical and Computational Chemistry, Institute of Theoretical Chemistry, Jilin University, Changchun, China; ^3^Department of Electric Engineering and Computer Science, C.S. Bond Life Sciences Center, University of Missouri, Columbia, MO, United States; ^4^College of Computer Science and Technology, Jilin University, Changchun, China

**Keywords:** *Thermus thermophilus* Argonaute, gene editing, CRISPR, molecular dynamics simulation, MM-PBSA, conformational change

## Abstract

*Thermus thermophiles* Argonaute (TtAgo) is a complex, which is consisted of 5′-phosphorylated guide DNA and a series of target DNA with catalytic activities at high temperatures. To understand why high temperatures are needed for the catalytic activities, three molecular dynamics simulations and binding free energy calculations at 310, 324, and 338K were performed for the TtAgo-DNA complex to explore the conformational changes between 16-mer guide DNA/15-mer target DNA and TtAgo at different temperatures. The simulation results indicate that a collapse of a small β-strand (residues 507–509) at 310 K caused Glu512 to move away from the catalytic residues Asp546 and Asp478, resulting in a decrease in catalytic activity, which was not observed in the simulations at 324 and 338 K. The nucleic acid binding channel became enlarged at 324 and 338K, thereby facilitating the DNA to slide in. Binding free energy calculations and hydrogen bond occupancy indicated that the interaction between TtAgo and the DNA was more stable at 324K and 338K than at 310 K. The DNA binding pocket residues Lys575 and Asn590 became less solvent accessible at 324 and 338K than at 310 K to influence hydrophilic interaction with DNA. Our simulation studies shed some light on the mechanism of TtAgo and explained why a high temperature was needed by TtAgo during gene editing of CRISPR.

## Introduction

Argonaute (Ago) proteins preform a critical role in guide target RNA recognition, cleavage, and product release, which depend on key components of a RNA-induced silencing complex (Peters and Meister, 2007; Hutvagner and Simard, 2008; Kawamata and Tomari, 2010; Sheng et al., 2014). These proteins belong to the TNRC6/GW182 protein family, coordinates downstream silencing events, which also named TNRC6A-C and GW proteins in humans. Ago proteins are provided with four domains with distinct functions (Huntzinger and Izaurralde, 2011; Pfaff and Meister, 2013; Chen et al., 2014; Mathys et al., 2014; Hauptmann et al., 2015). The N-terminal domain plays a key role for small RNA binding. The P element-induced wimpy testis (PIWI)–Argonaute–Zwille (PAZ) domain is essential for recruiting the middle domain (MID) and the 5′ end of a small RNA. The PIWI domain has the same function as the RNase H domain, and some Ago proteins can serve as small RNA-guided endonucleases (Hauptmann et al., 2015). The PIWI domain is essential for the cleavage activity of Ago in which the Asp–Asp–Asp/His catalytic triad of Ago processes the identification of double stranded RNAs and the cleaving of their strand (Liu et al., 2004; Song et al., 2004; Ma et al., 2005; Rivas et al., 2005; Yuan et al., 2005; Parker and Roe, 2014). It also acts on guide-target RNA duplexes to cleave their target strand. Mg^2+^ cation mediates the endonucleolytic cleavage of a target RNA strand (Martinez and Tuschl, 2004; Schwarz et al., 2004; Lingel and Sattler, 2005; Jinek and Doudna, 2009; Parker, 2010), thereby forming 3′-OH and 5′-phosphate ends (Elbashir et al., 2001). A series of experimental structures of *Thermus thermophilus* Argonaute (TtAgo) binding to various complexes of different nucleic acid strands provide insights into conformational changes in proteins and DNAs (Jung et al., 2013; Zhu et al., 2016a,b; Sheng et al., 2017). Two structures, namely, PDB ID 3F73 and PDB ID 3HM9, whose substrates have different lengths (Wang et al., 2008a,b), suggested that the PAZ domain undergo a series of motions during a catalytic cycle. They also reveal the PAZ domain disassociates from the 3′ end of the guide upon target binding (Xia et al., 2012; Wang et al., 2013; Nam et al., 2014; Swarts et al., 2014; Jiang et al., 2015).

**Figure 1:**
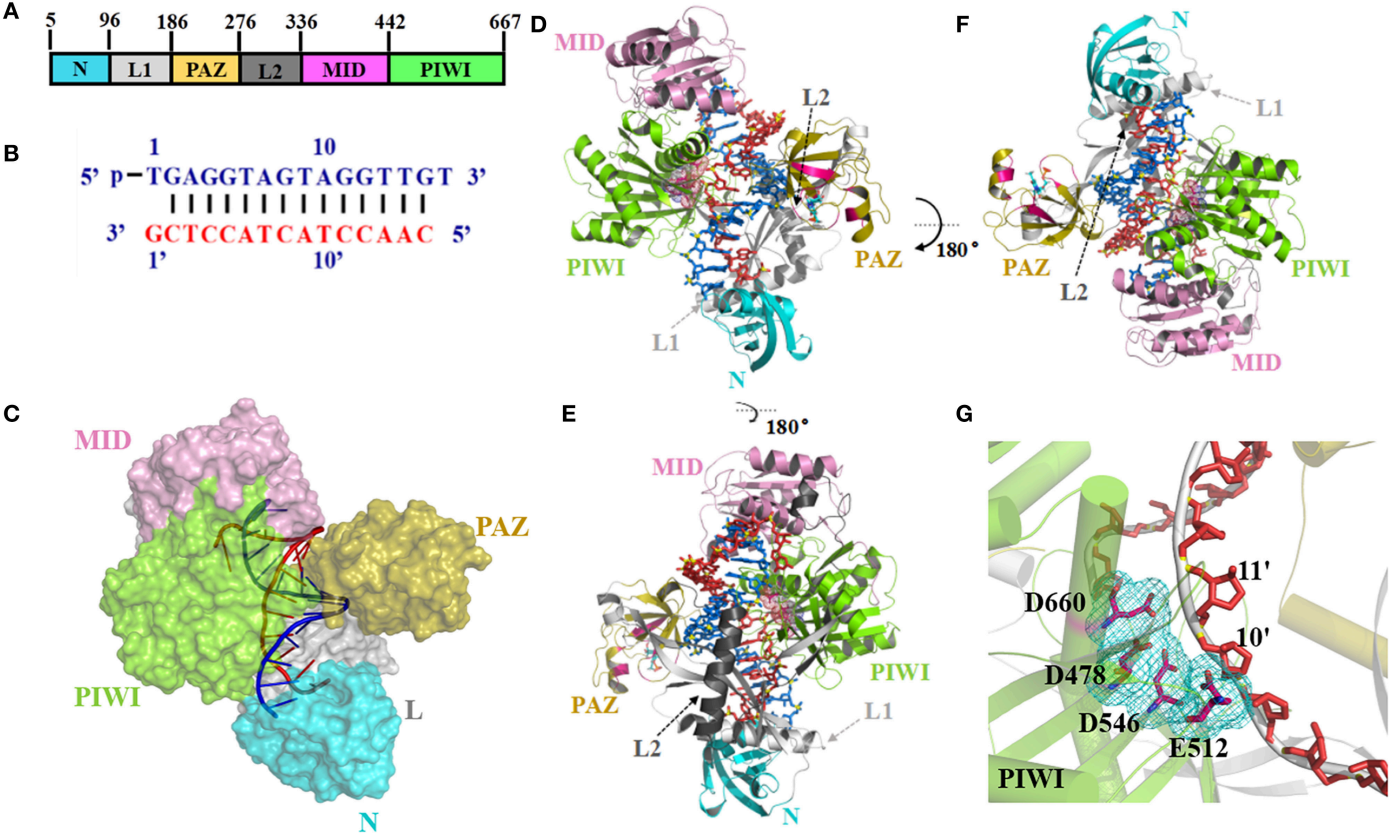
Argonaute protein of TtAgo. **(A)** Domain composition (left) and nucleic acid binding channel (right) of TtAgo (PDB 4NCB). **(B)** Sequence and pairing of guide (blue) and target (red) strands in the ternary complex of 4NCB. **(C–E)** The 3-D structures of TtAgo ternary complex were colored as **(A,B)**. In **(C)**, the TtAgo protein is shown as a surface, whereas the nucleic acid is shown as a cartoon. In **(D,E)**, the TtAgo protein is shown as a cartoon, whereas the nucleic acid is shown as a stick. **(G)** Catalytic residues of TtAgo. The catalytic residues in a stick representation were highlighted by pink sticks and a cyan mesh.

This study focused on TtAgo. Figure 1 shows the crystal structure of TtAgo (PDB ID 4NCB) with bound 16-mer guide DNA and 15-mer target DNA (Sheng et al., 2014). TtAgo is also made up by four functional domains, similar to other Ago proteins, which named N, PAZ, MID and C-terminal PIWI domain. These four domains are connected by two linker domains L1 (Linker 1) and Linker 2 (L2) (Figures 1A–F). MID bind to 5′ terminals of the guide and PAZ bind to 3′ terminals of the guide, both of them define the nucleic acid binding channel (Figure 1C and Figure S1). Driven mainly by positively charged residues of TtAgo and negatively charged DNA backbone atoms (Zander et al., 2014; Swarts et al., 2015; Figure S1A), the nucleotides 2–8 of the guide of 5′ segment, which called seed region, involved the combination of the channel binding (Figures S1B,C). The correct positioning of the seed region is important for the binding between the target nucleic acid and TtAgo and human Ago (Lee et al., 2016; Khin et al., 2017). The distance between Glu512 and active center (Asp478, Asp546, and Asp660) directly affects the activity of TtAgo. When Glu512 is far from the catalytic pocket (Figure S1D; Wang et al., 2008a,b), it turns out to be a cleavage-incompatible conformation. Glu512 positioned on loop L2, close to the catalytic pocket, represents a key residue for a cleavage-compatible conformation (Figure 1G and Figure S1E).

As CRISPR/Cas9 becomes a popular molecular biology technology for gene editing, there is a continuous interest in searching for new genome-editing methods. Recently, it has been reported that argonaute proteins prossess DNA-guided sequence-specific DNA endonuclease activity in *Pyrococcus furiosus, Thermus thermophilus*, and *Methanocaldococcus jannaschii* (Beshnova et al., 2014; Cherstvy and Teif, 2014; Harikrishna and Pradeepkumar, 2017) and it can be as a host defense system to eliminate invading nucleic acids. These argonaute proteins usually needs high temperature to be fully active, which may restrict the application in many aspects. In 2016, the argonaute protein (NgAgo) from *Natronobacterium gregoryi* was reported to as mesophilic microbe whose enzymes, which is functional at 310K. Compared to Cas9, it have higher genome editing activity in mammalian cells (Chandradoss et al., 2015). Since NgAgo does not require a PAM motif and the authors reported the PAM motif is not essential for NgAgo and high efficiency to targets of high guanine-cytosine content, NgAgo was once considered promising as a useful tool for genome editing. However, as soon as researchers set off on a whirlwind of experiments (Chandradoss et al., 2015; Salomon et al., 2015), questions started to emerge and the hope was quickly faded. Nevertheless, it is unclear why NgAgo cannot perform the function of TtAgo, although the two proteins have significant sequence similarity, and whether it is possible to use or modify argonaute as a target for gene editing.

It has been reported that TtAgo is active at high temperatures, not at low temperatures. But how temperature affects TtAgo activity, it has not been clarified so far. A few molecular dynamics (MD) studies on siRNA–TtAgo and siRNA–hAGO2 complexes have been conducted (Wang et al., 2013; Nam et al., 2014; Hanlun et al., 2015). Jiang et al. constructed from MD simulations and found that MSMs can elucidate the conformational dynamics of AGO at biologically relevant timescales (Hanlun et al., 2015). Jinhyuk Lee pointed out that they revealed different structural conformations of the RNA duplex in Ago2, depending on Mg^2+^concentration and demonstrated that cation effects on Ago2 structural flexibility are critical to its catalytic/functional activity, with low Mg^2+^ favoring greater Ago2 flexibility and less miRNA/mRNA duplex stability, thus favoring slicing (Nam et al., 2014). Wang et al. discovered three findings during MD simulations: (1) three important (PAZ, Mid and PIWI) domains existed in Argonaute which defined the global dynamics of the protein; (2) the interdomain correlated movements were so crucial for the interaction of Ago-RNAs that they not only facilitated the relaxation of the interactions between residues surrounding the RNA binding channel but also induced certain conformational changes; and (3) it was just these conformational changes that expand the cavity of the active site and open putative pathways for both the substrate uptake and product release (Wang et al., 2013). However, several fundamental questions remain unanswered: (1) How does the temperature affect the catalytic activity of Ago? (2) What factors cause the conformational changes in the catalytic center at different temperatures?

In the present work, MD simulations and binding free energy calculations (molecular mechanics–Poisson–Boltzmann surface area, MM-PBSA) were performed on 16-mer guide DNA, 15-mer target DNA, and TtAgo at temperatures of 310, 324, and 338K to find possible answers to these questions. Principal component analysis (PCA) was also applied to investigate the interactions between TtAgo and DNA. Our MD results provided some informative clues for the mechanism of conformational changes in the catalytic center at different temperatures.

## Materials and methods

### Protein preparation

In 2014, Sheng et al determined the crystal structures of ternary *Thermus thermophilus* Argonaute (*Tt*Ago) complexes with 5′-phosphorylated guide DNA and a series of DNA targets (PDB Id 4NCB) (Sheng et al., 2014), in which the complex structures solved at improved resolution up to 2.2 Å. It was very interesting reliable target for MD simulation. And so we chose *Tt*Ago (PDB Id 4NCB) to perform simulations. The initial structure of TtAgo–nucleic acid complex in the MD simulations was obtained from the Protein Data Bank (PDB ID 4NCB at 2.19 A resolution). TtAgo is a 685-amino acid protein, and its binding nucleic acid is composed of 16-mer guide DNA and 15-mer target DNA. The Mg^2+^ in the 4NCB structure were also used in the simulations.

### Conventional MD simulations and analysis

The three systems with different temperatures (310, 324, and 338K) were utilized in 200 ns MD simulations with NAMD2.10 b1 by using CHARMM27 all-force field parameters (Best et al., 2012). In TtAgo, for Mg^2+^ and nucleic acids (guide DNA and a series of DNA targets), generalized CHARMM27 all-force field parameters (Mackerell et al., 2004) were applied. In three system, the complexes were placed in the explicit TIP3P water model (Jorgensen et al., 1998), and extended at 15 Å from water molecules in a cubic periodic box. The systems were added with sodium and chloride ions to obtain a final ion concentration of 0.15 mol/L. To avoid steric clashes or improper geometries, the steepest descent algorithm with 50,000 steps was carried out to was energetically minimized for each system. The Langevin dynamics (Schlick, 2002) was used to constant temperature control with a damping coefficient (gamma) of 1.0 ps. The long-range electrostatic interactions was calculated by Particle Mesh Ewald summation algorithm (Darden et al., 1993). Each simulation was performed for 200 ns for the protein–nucleic acid complexes at different temperatures (310, 324, and 338K) by using a pressure coupling time constant of 2.0 ps and a temperature coupling time constant of 0.1 ps and a pressure coupling time constant of 2.0 ps. The timestep for each simulation was 1ps.

### Cluster analysis

Clusteringis a general data-mining technique (Cormack, 1971; Jain et al., 1999) using different methods (algorithms) that can be applied to judge structure similarity. In this study, clustering was performed on three systems (the 310K, 324K, and 338K) molecular dynamics simulation trajectories using the RMSD as a metric according to hierarchical method (Rokach and Maimon, 2005) into distinct groups. The cut off value of RMSD was set with 2.0Å for each systems. Then the TtAgo structure in the 200 ns simulation trajectories which had the similar conformation were divided into the same cluster. The proportion of each cluster were calculated and representative frame of the two largest clusters were extracted for the further analysis.

### Binding free energy calculation

The Equation (1) was used to calculate the binding free energy as follows:

(1) $$\Delta {\text{G}}_{\text{b}}=\Delta {\text{G}}_{\text{int}}^{\text{ele}}+\Delta {\text{G}}_{\text{int}}^{\text{vdw}}+\Delta {\text{G}}_{\text{sol}}-T\Delta \text{S}$$

where ΔG_b_ is the total binding free energy; The van der Waals interaction energies and electrostatic between a protein and its ligand are Δ${\text{G}}_{\text{int}}^{\text{ele}}$ and Δ${\text{G}}_{\text{int}}^{\text{vdw}}$, respectively; TΔS is the contribution of conformational entropy to the binding; ΔG_sol_ is the solvation energy, and. Δ${\text{G}}_{\text{int}}^{\text{ele}}$ and Δ${\text{G}}_{\text{int}}^{\text{vdw}}$ were calculated by the same parameter set as in the molecular dynamics simulation, but no cutoff was applied to the calculation.

The binding free energies of nucleic acids and TtAgo in different temperature systems was performed by MM-PBSA calculation. The Amber 14 package was used to perform 2 ns simulation with the Amber ff99 force field parameters (Kollman et al., 2000). In our study, 500 snapshots were extracted from the last 1.5 ns MD trajectory for all of the energy components calculating.

## Results and discussion

### Stability of TtAgo in different temperature systems

**Figure 2:**
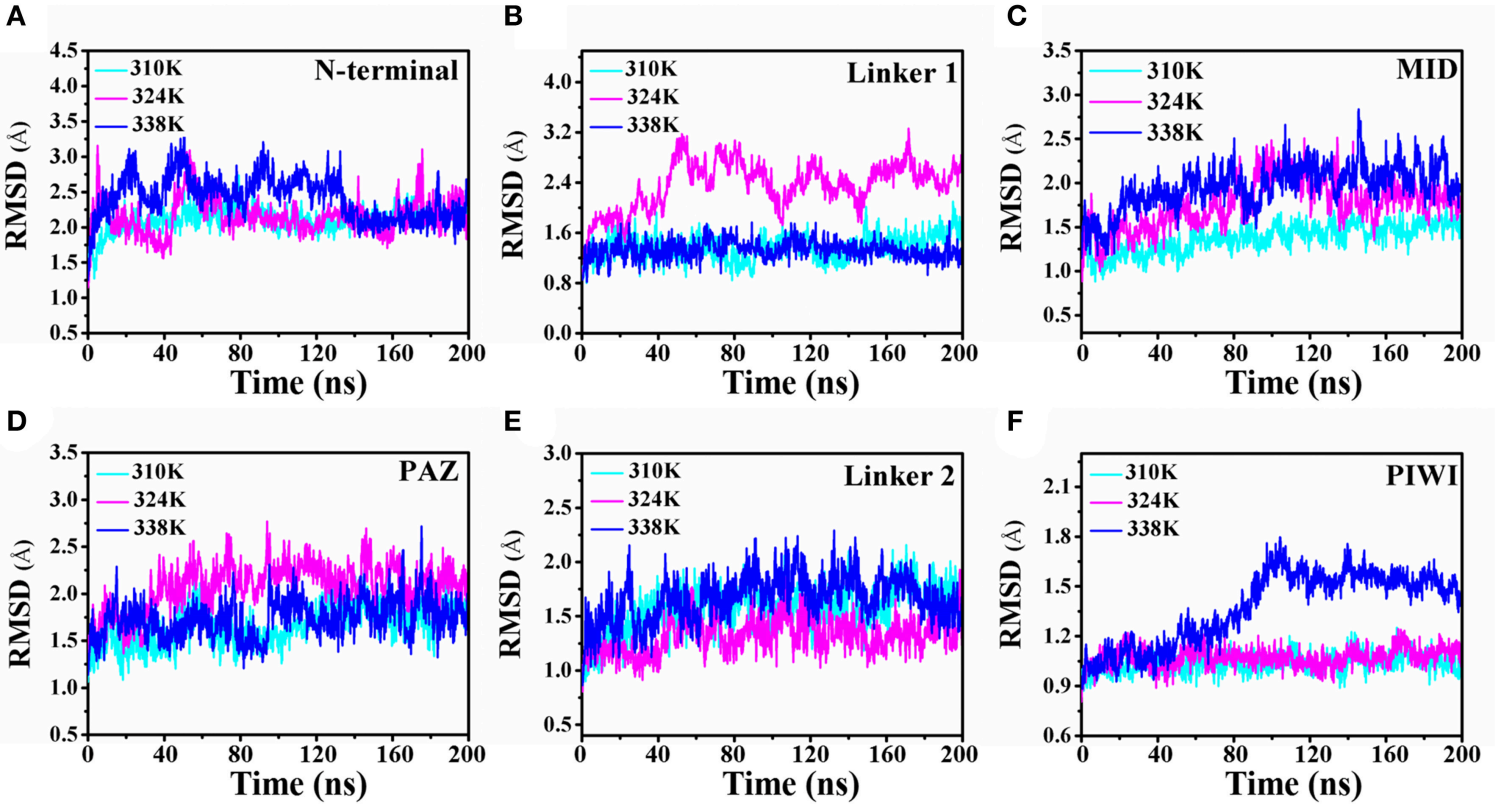
Comparison of the RMSD of the 310K, 324K, and 338K systems at different TtAgo domains. **(A–F)** RMSD plot of backbone atoms in different domains in three temperature systems.

To analyze the influence of temperature on TtAgo activity, the 200 ns molecular dynamics simulation prepared at three temperatures (310, 324, and 338K) were prepared and the simulation of each temperature was repeated three times. The root mean square deviation (RMSD) was monitored with the three different temperature systems of MD trajectories to measure the stability of each domain of TtAgo with temperature variation and to examine the protein stability during the MD simulations. The RMSD of protein backbone atoms and different domain backbone atoms was conducted (Figure 2 and Figure S2). In Figure S2, the RMSD variations of the different temperature systems for three times at the 200 ns time scale indicated that the complexes had the similar atomic coordinates and the initial structures. The RMSDs of the 310, 324, and 338K systems converged to 1.80, 2.40, and 2.20 Å, respectively, indicating that the three systems were stable. The RMSDs of different domains in the three systems are shown in Figures 2A–F. The Linker 1 domain of TtAgo became more flexible in the 324K system (Figure 2B) than that of the 310 and 338 K systems, whereas the PIWI domain was more flexible in the 338K system than in the two other systems (Figure 2F). The increase in temperature causes the protein residues to fluctuate dramatically. In this study, rising the temperature to 324K and 338K resulted in an increase of flexible for Linker 1 or PIWI domains. This may affect the relative position between the Linker 1 and PIWI and thus regulated the catalytic activity of TtAgo. In Figure S3, the total energy vs. time was also calculated and it can be seen that the total energy for three systems keep steady during 200 ns simulation. In addition, We chose the four structures with lower energy from three simulation trajectories and calculated the Ramachandran Plot. As the Figures S4–S6. The four lower energy structures for each system had the same Ramachandran conformation which indicated that the whole simulation process was stable.

**Figure 3:**
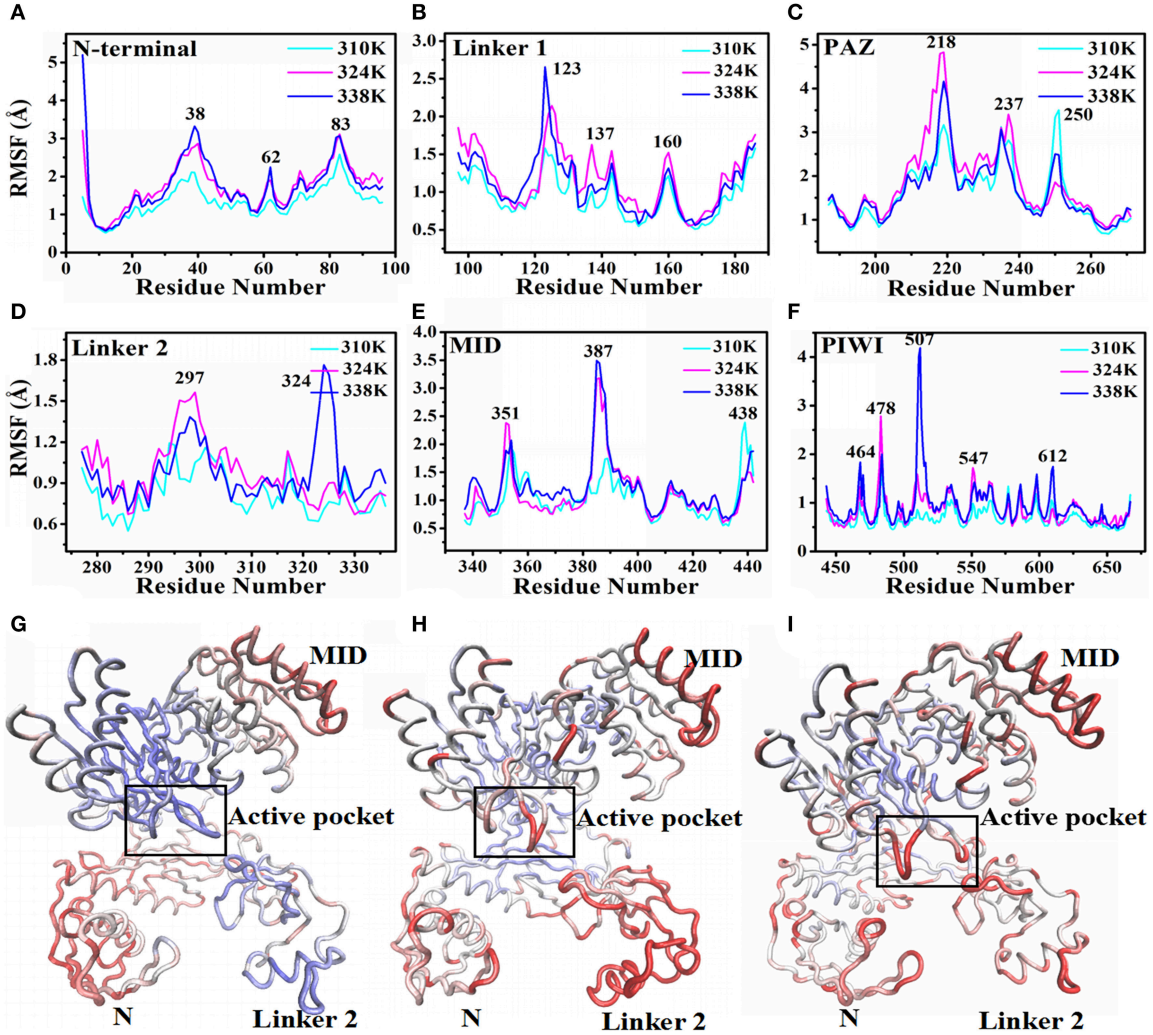
RMSF and backbone flexibility during 200 ns MD at different TtAgo domains. **(A)** RMSF values of the N terminus calculated in three temperature systems for 200 ns MD trajectories. **(B)** RMSF values of Linker 1 calculated in three temperature systems for 200 ns MD trajectories. **(C)** RMSF values of PAZ calculated in three temperature systems for 200 ns MD trajectories. **(D)** RMSF values of Linker 2 calculated in three temperature systems for 200 ns MD trajectories. **(E)** RMSF values of MID calculated in three temperature systems for 200 ns MD trajectories. **(F)** RMSF values of PIWI calculated in three temperature systems for 200 ns MD trajectories. **(G–I)** Visualizations of backbone flexibility of TgAgo at 310 K **(G)**, 324K **(H)**, and 338 K **(I)**. The different degrees of motion correspond to tubes with different colors and thicknesses.

Then the root-mean-square fluctuation (RMSF) for the backbone atoms of the nucleic acids with TtAgo protein in different temperature systems was analyzed to determine the mobility of the complexes. Figure 3 illustrates that the large punctuations of residues mainly occurred in the 324 and 338K systems. The RMSF fluctuations of Gly38, Asp62, and Gly83 located at the N terminus (Figure 3A), Arg123 and Arg137 located at Linker1 (Figure 3B), Pro218 located at PAZ (Figure 3C), Ile297 and Arg324 located at Linker 2 (Figure 3D), Gln387 located at MID (Figure 3E), and Asp478, Glu507, Gly547, and Gly612 located at PIWI (Figure 3F) were higher in the 324 and 338K systems than in the 310 K system. The results indicated that the protein at 324 and 338K exhibited a higher mobility than at 310 K during MD simulations. Asp478, Glu507, and Gly547 near the active center at the PIWI domain that exhibited a high mobility in the 324 and 338K systems might affect the catalytic activity of TtAgo. To further confirm the RMSF results, we calculated the RMSD values for each residue, and then colored each according to the RMSD value (Figures 3G–I). It can be seen that the intensity of the movement of various residues in the 324 or 338K system was higher than that in the 310K system, especially in the Linker 2 and active pocket domain. In order to further analyze residual atomic flexibility, an isotropic temperature factor (B-factor) has been calculated and the results was shown in Figure S7. As we can see, the change trend of B-factor was consistent with that of RMSF and RMSD of individual residues. Figure S8 shows that the radius of gyration (Rg) in different domains in the three systems during the 200 ns time scale MD was stable.

The solvent-accessible surface area (SASA) predicts the number of residues present in the outlier regions (surface) of the protein and the number of residues that are in the hydrophobic core (buried). In this study, the time-dependent SASA in the different domains of TtAgo from the simulations was also calculated (Figure S9). The results showed that the ensembles of the different domains in the three temperature systems were similar, which indicated that the change in temperature hardly influenced the SASA value of each domain. This analysis further calculated the SASA values of the residues that interact with the guide DNA and target DNA. As we can see in Figure S10A, the SASA of the hydrophilic residues Lys575 and Asp590 decreased in the 324 and 338K systems compared with that in the 310K system, thereby illustrating increased interaction with the DNA. Asp590 interacted with the G1 of the target DNA (Figure S10B), whereas Lys575 interacted with the C11 and C12 of the target DNA (Figure S10C), forming a tighter interaction with DNA. This result indicated that TtAgo at 324 and 338K might have undergone drifting, thereby leading to its transition into an advantageous conformation and binding with a nucleic acid.

### Conformational changes in different temperature systems

**Figure 4:**
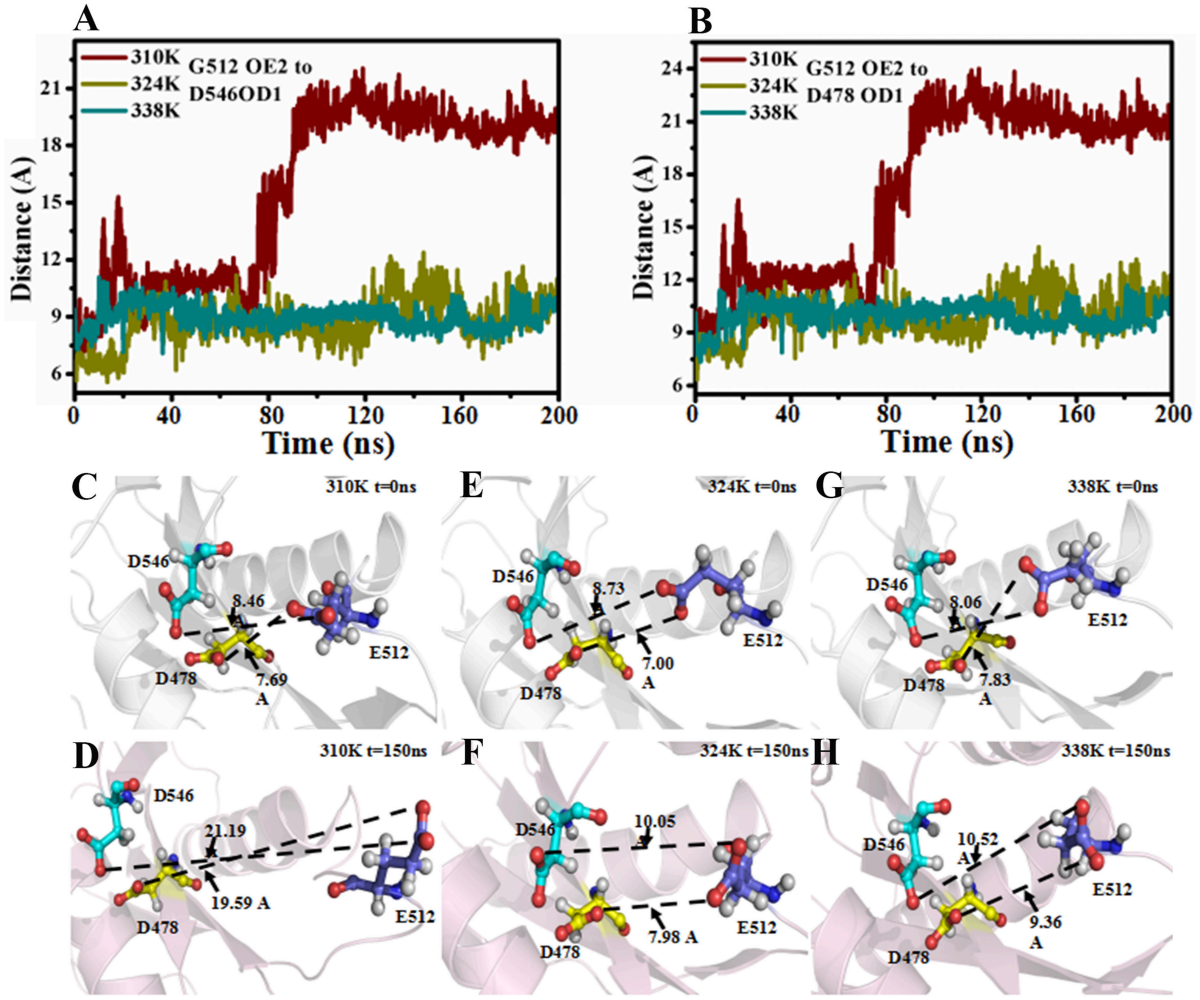
Distance between Glu512 and active pocket in 310K, 324K, and 338K systems. **(A)** Time evolution of distance between Glu512 OE2 and Asp478 OD1 in three temperature systems. **(B)** Time evolution of distance between Glu512 OE1 and Asp546 OD1 in three temperature systems. **(C,D)** Distance between Glu512 (blue) and active pocket (Asp546, cyan; Asp478, yellow) in 310K system at 0 and 150 ns. **(E,F)** Value of distance between Glu512 (blue) and active pocket (Asp546, cyan; Asp478, yellow) in 324K system at 0 and 150 ns. **(G,H)** Distance between Glu512 (blue) and active pocket (Asp546, cyan; Asp478, yellow) in 338K system at 0 and 150 ns.

**Figure 5:**
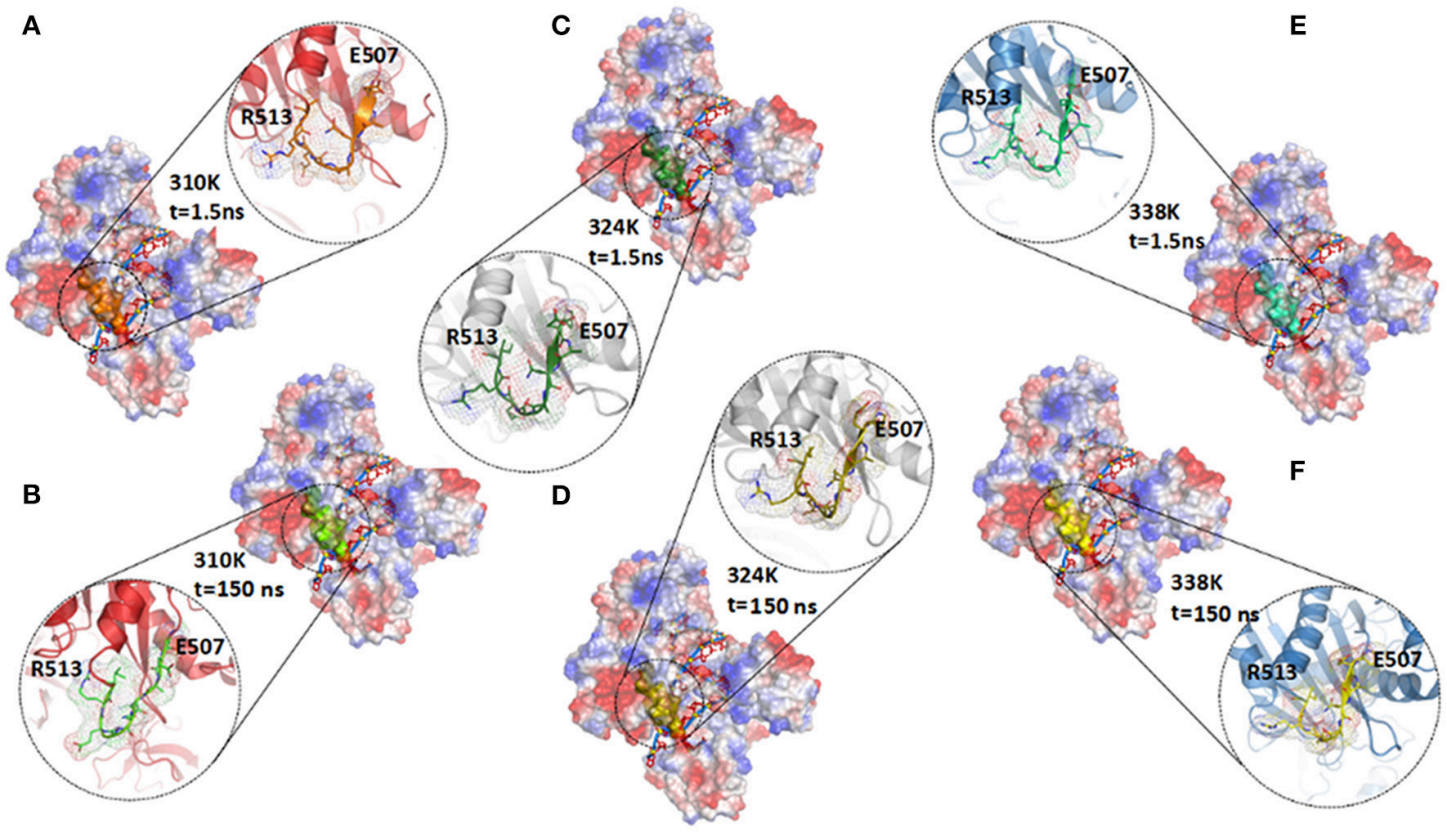
Comparison of secondary structure in different temperature systems. **(A,B)** Structure of TtAgo in 310K system at 1.5 ns **(A)** and 150 ns **(B)** during 200 ns MD, and residues Glu507 to Arg513 were highlighted with orange (1.5 ns) and green (150 ns) cartoons. **(C,D)** Structure of TtAgo in 324K system at 1.5 **(C)** and 150 ns **(D)** during 200 ns MD, and residues Glu507 to Arg513 were highlighted with split-pea (1.5 ns) and yellow (150 ns) cartoons. **(E,F)** Structure of TtAgo in 324K system at 1.5 **(E)** and 150 ns **(F)** during 200 ns MD, and residues Glu507 to Arg513 were highlighted with cyan (1.5 ns) and yellow (150 ns) cartoons.

It has been reported that the binary complex of a yeast Ago with a bound 5′-phosphorylated guide RNA strand have found that the distance between Glu512 and catalytic pocket composed of three Asp residues, namely, Asp478, Asp546, and Asp660, determines the activity of Ago (Nakanishi et al., 2012). The activated Ago needs to insert Glu512 into the catalytic pocket. The change in the distance between Glu512 and the catalytic pocket during MD simulations in the three temperature systems was also analyzed to observe the influence of temperature change on enzyme catalytic activity. In Figure 4A, the distance between Glu512 and Asp478 in the 310K system increased from 10 to 21 Å at 80 ns. The distance between Glu512 and Asp546 in the 310K system also increased from 9.50 to 23 Å at 80 ns (Figure 4B). In the 324 and 338K systems, the distance from Glu512 to Asp546 and Asp478 was approximately 10 Å during MD simulations. In addition, the distance between Glu512 and Asp478 or Asp546 for other two repeated simulations were calculated and the results were prepared in Figure S11. It can be seen that the distance between Glu512 and Asp478 or Asp546 showed a remarkably increase at 47 and 90 ns in 310K system respectively. But for the 324 and 338K systems, the distance from Glu512 to Asp546 and Asp478 was approximately 10 Å during two MD simulations. This finding implied that the distance from the Glu512 to the active center in the 310K system was larger than that in the 324 and 328K systems. To confirm these results, we compared the structures between 0 and 150 ns at different temperatures (Figures 4C–H).The distance from Glu512 OE2 to Asp546 OD1 and Asp478 OD1 at 150 ns was 2.5 times than at 0 ns in the 310K system (Figures 4C,D). The distance between 0 and 150 ns changed slightly in the 324 and 338K systems compared with that in the 310 K system (Figures 4E, 5H). To illustrate the dynamic process of distance change, we created animations 1–3 in Supporting information of the distance between the Glu512 OE2 and Asp478 OD1 of the visual dynamic variation at three different temperatures. The results revealed that Glu512 was far from the catalytic pocket at 310K, and the enzyme activity at 324 and 338K was stronger than that at 310K.

To explain the cause of the change in distance, we investigated the secondary structure modifications during 200 ns MD simulations in the different temperature systems (Figures S12A–C and Figure 5; Table 1). The β-strand of Glu507 to Gln509 near Glu512 disappeared at 150 ns compared with that at1.5 ns in the 310K system (Figure S12A). The β-strand contents of Glu507 to Gln509 in the 310, 324, and 338K systems were 1.50, 37.60, and 46.80%, respectively (Table 1, the β-strand contents of Glu507 to Gln509 in the 310, 324, and 338K systems for other two repeated simulations were calculated and the results were prepared in Tables S1, S2). Secondary structure analysis revealed that the short intermediate β-sheet at 324 and 338K during 200 ns molecular dynamics simulation represented the ordered structure that might help maintain the close distance between Glu512 and the catalytic pocket. Glu512 moved far from the catalytic pocket when β-sheet disappeared.

**Table 1:** Probability of generating β-bridge in E512 loop and D546 loop.

**System**	**E512 loop**	**B-brige occupancy**
310K	E507 to Q509	0.5%
324K	E507 to Q509	37.6%
338K	E507 to Q509	46.8%

### Changes in nucleic acid binding channel in different temperature systems

**Figure 6:**
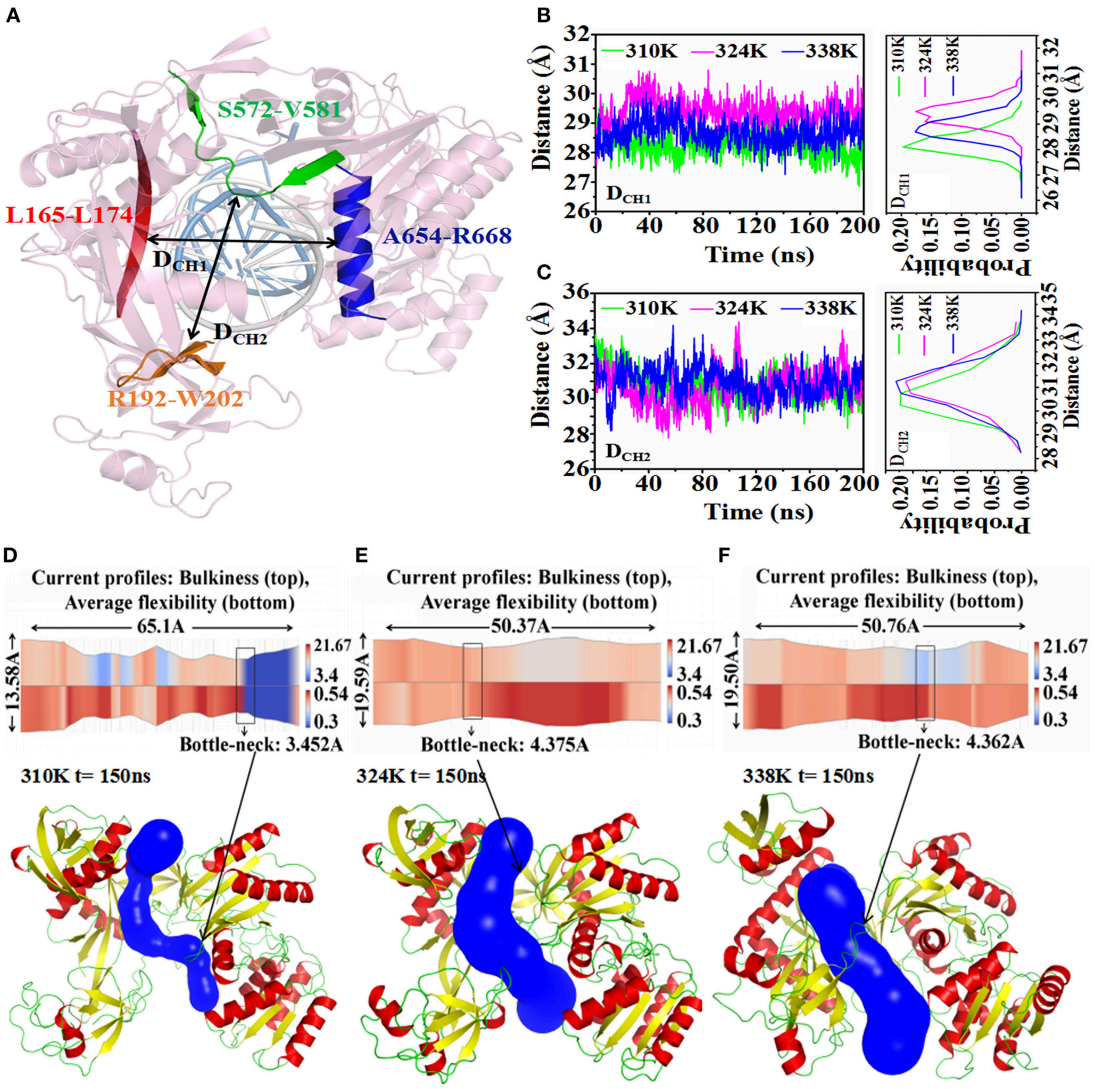
Analysis of nucleic acid binding channel in three temperature systems. **(A)** D_CH1_ and D_CH2_ were used to describe the diameter of the nucleic acid binding channel. D_CH1_ measured the c.o.m. distance in residues 165–174 (red cartoon) and in residues 654–668 (blue cartoon). D_CH2_ was the c.o.m. distance in residues 192–202 (orange cartoon) and in residues 572–581 (green cartoon). The guide-DNA and target-DNA were highlighted by white and marine cartoon respectively. **(B)** Time series of D_CH1_ in three temperature systems (left) and the probability distribution of D_CH2_ calculated from 0 to 200 ns trajectories (right). **(C)** Time series of the value for D_CH2_ in three temperature systems (left) and the probability distribution of D_CH2_ calculated from 0 to 200 ns trajectories (right). **(D–F)** 2-D(top) and 3-D (bottom) visualization of the channel obtained with CHEXVIS in the 310K **(D)**, 324K **(E)**, and 338K systems **(F)** for 150 ns structure. For 2-D channel profile visualization, bulkiness and average flexibility are shown in split visualization over the radius profile as a color map. The bottleneck radius was highlighted by rectangles. For 3-D channel profile visualization, the channel was highlighted with blue, and TtAgo was represented by a cartoon.

The minimum distances from Linker 1 to PIWI and from PAZ to PIWI have been reported to determine the nucleic acid binding channel in TtAgo. In our study, D_CH1_ was used to describe the center of mass (c.o.m.) distance from the part of L1 (residues 165–174, highlighted in red) to the long helix on PIWI (residues 654–668, highlighted in blue). D_CH2_ was the c.o.m. distance from the part of PAZ (residues 192–202, highlighted in pink) to the part of PIWI (residues 572–581, highlighted in green), as shown in Figure 6A. These two distances were used to measure the diameter of the nucleic acid binding channel. To analyze the effect of temperature change on the nucleic acid binding channel, we detected the changes in D_CH1_ and D_CH2_ during 200ns MD in the different temperature systems (Figures 6B,C). D_CH1_ varied at 27.5, 28.5, and 29.5 Å in the 310, 324, and 338K systems, respectively. These findings suggested that the minimum distance from Linker 1 to PIWI was larger in the 324 and 338K systems than that in the 310K system. Similarly, D_CH2_ reached 31 and 30 Å in the 324 and 338K systems and in the 310K system, respectively.

CHEXVIS is a tool used for molecular channel calculation and visualization based on alpha complex representation (Masood et al., 2015). In addition to predictive channels, channel residues and their flexibility can be predicted by CHEXVIS. In our study, this tool was utilized to analyze the distinction of the nucleic acid binding channels in the different temperature systems at 150 ns for 200 ns MD (Figures 6D–F; Table 2). The length of the channel in TtAgo in the 310K system (60.1Å) was longer than those in the 324 (50.37Å) and 338K (50.76Å) systems. Studies on bottlenecks have recommended promising areas for the modification of channel properties because their substitutions can affect the informative tunnel geometry when protein dynamics is considered. The results showed that the bottleneck of TtAgo in the 310K system was 3.452 Å and changed to 4.375 and 4.362 Å in the 324 and 338K systems, respectively. The analysis of bulkiness and average flexibility of channel residues, which were presented in a 2D profile, suggested that the flexibility of the channel residues was higher in the 324 and 338K systems than in the 310K system. With such a high flexibility, the channels easily become wide. These results indicated that the diameter of nucleic acid binding channels were larger and the binding of nucleic acid and protein may occur more easily in the 324 and 338K systems than in the 310K system as temperature increased. Our theoretical finding is consistent with the computational study made by Wang et al. (2013).

**Table 2:** Nucleic acid binding channel comparison for different temperature systems.

**Channel**	**Length(A)**	**Bottle-neck(A)**
310K system	65.10	3.452
324K system	50.37	4.375
338K system	50.76	4.362

### Cluster analysis

**Figure 7:**
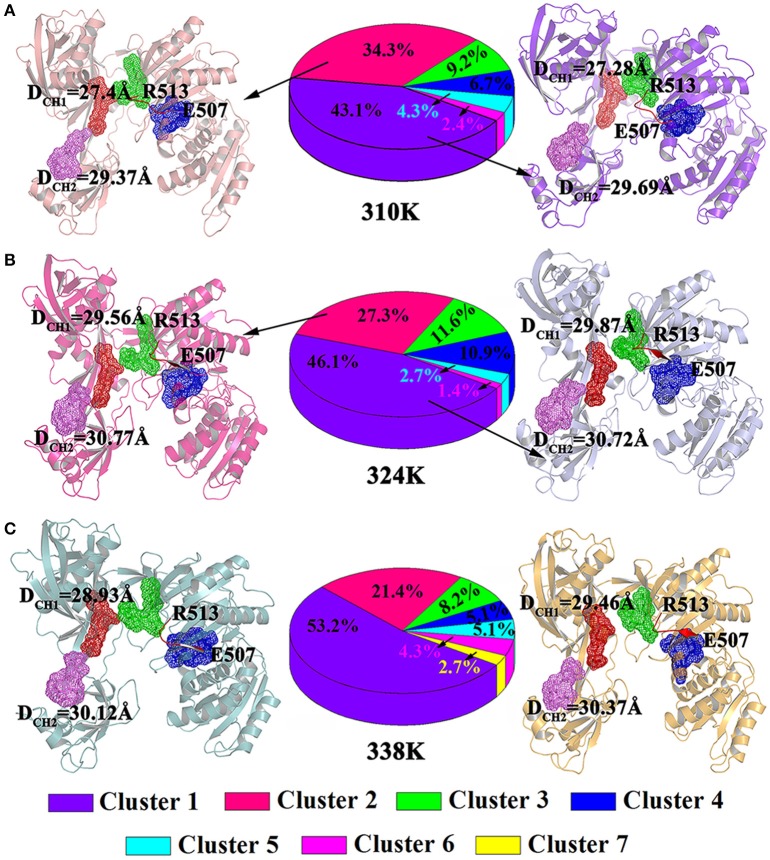
Analysis on clusters in the 310K **(A)**, 324K **(B)**, and 338K systems **(C)**. Two representative structures of most populated clusters in three systems are represented as cartoons. The D_CH1_ and D_CH2_ residues were highlighted with mesh. The beta strands at residues Glu507 to Arg513 were colored in red, while the loops at residues Glu507 to Arg513 were colored by orange.

In addition, clusteranalysis was performed on each trajectory of the three systems to reveal a clear-cut structural difference in the nucleic acid and TtAgo complex in the different temperature systems. As shown in Figure 7A, conformations for 310K systems during the simulations were divided into six clusters and the percentage population of clusters was 43.1, 34.3, 9.2, 6.7, 4.3, 8, 2.4, 5, and 4% respectively. Then the representative structures of most populated cluster in cluster1 and cluster2 were selected to analyze the conformation and channel. It can be seen that the residues Glu507 to Gln509 in both the representative structures of cluster1 and cluster2 at 310K formed a loop. The D_CH1_ and D_CH2_ in cluster1 representative structures were 27.43 and 29.37Å while they were 27.28 and 29.69Å in cluster2 representative structures, respectively.

Figures 7B,C exhibited the cluster analysis for 324K and 338K systems, respectively. The percentage population of cluster1 and cluster2 for 324K and 338K systems were 46.1, 27.3, 53.2, and 21.4%, respectively. The sum of cluster1 and cluster2 both in those two systems were over 70%. Two representative structures based clustering approach at 324K (cluster1 and cluster2) were extracted the results were shown in Figure 7B. In those conformations, the residues Glu507 to Gln509 remained β-sheet and the D_CH1_ were over 29.56Å in two representative structures while the and D_CH2_ were over 30 Å. As the same as 324K systems, residues Glu507 to Gln509 in cluster1 and cluster2 of 338K systems for representative structures also presented β-sheet. The D_CH1_ and D_CH2_ at 338K were more than 29 Å (Figure 7C). These findings revealed that the β-sheet (Glu507 to Gln509) at 310K formed a loop but remained in its original state in the 324 and 338K systems. These results were consistent with those illustrated in Figure 6. The different nucleic acid binding channels in the three temperature systems in the cluster1 and cluster2 of TtAgo illustrated that D_CH1_ and D_CH2_ in the 310K system were lower than those in the 324 and 338K systems.

### Hydrogen bond network analysis

**Figure 8:**
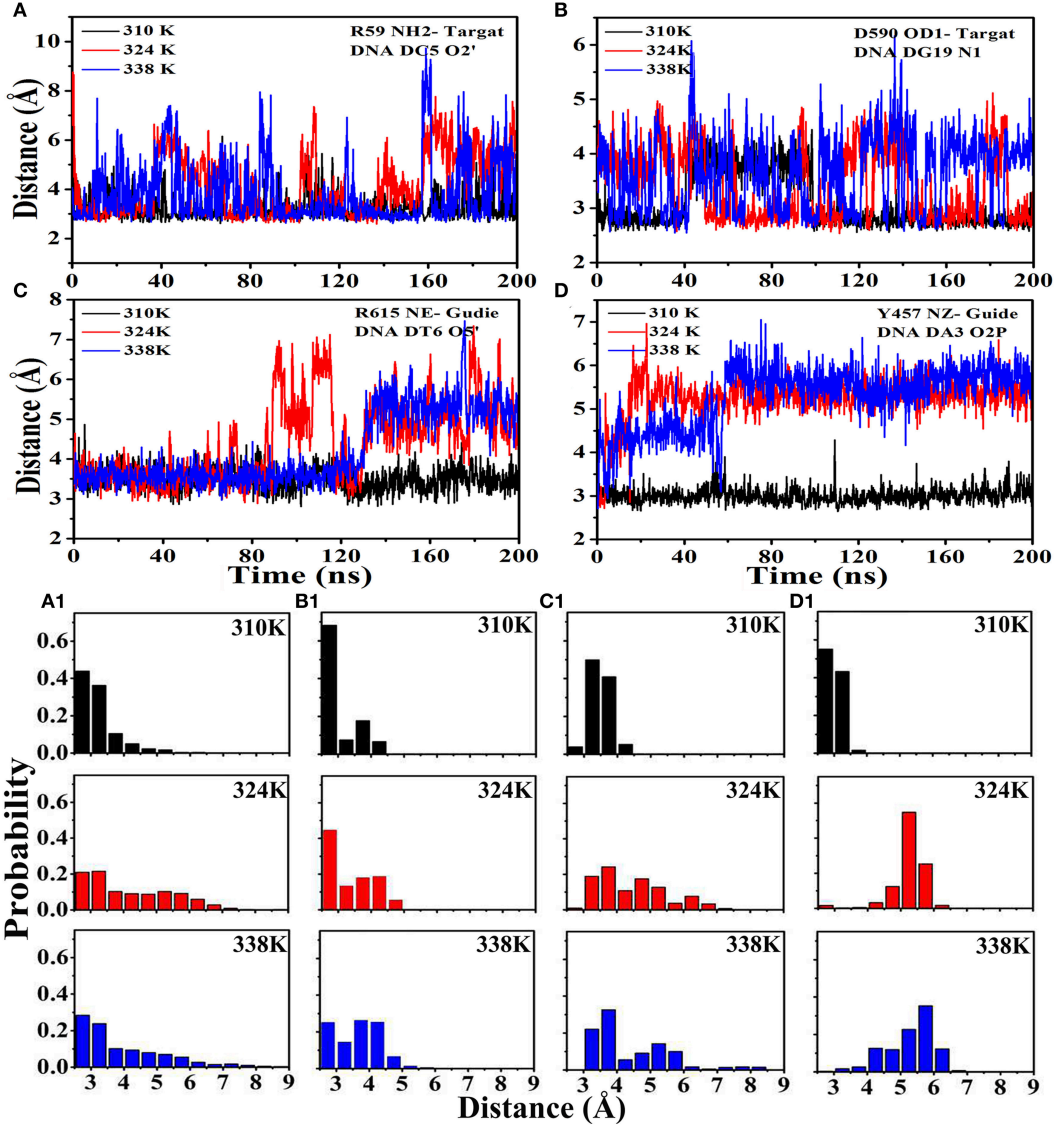
Distance between hydrogen bond donors and receptors at different temperatures. **(A)** Distance from R59 (HN2) to DC5 (O2) in the three systems and the probability exhibited in **(A1)**. **(B)** Distance from D590 (OD1) to DG19 (N1) in the three systems and the probability exhibited in **(B1)**.**(C)** Distance from R615 (NE) to DT6 (O5) in the three systems and the probability exhibited in **(C1)**. **(D)** Distance from Y457 (NZ) to DA3 (O2P) in the three systems and the probability exhibited in **(D1)**.

The hydrogen bond occupancy between nucleic acids (target DNA and guide DNA) and TtAgo during the 200 ns MD is listed in Tables S3, S4 to obtain insights into the molecular interactions between TtAgo and nucleic acid at different temperatures. The MD simulation results showed that the number of hydrogen bonds between nucleic acids and TtAgo in the 310K system was more than that in the 324 and 338K systems. For example, the hydrogen bonds, R59 (HN2)—DC5 (O2), D590 (OD1)—DG19 (N1) that connected the protein and the target DNA, and R615 (NE)—DT6 (O5′) and Y457 (NZ)—DA3 (O2P) that linked TtAgo and guide DNA disappeared in the 324 and 338K systems. The decrease in hydrogen bond occupancy was due to the increase in the distance between hydrogen bond donors and receptors. The distances of the donors and receptors were examined during 200 ns MD (Figure 8). In Figure 8, the distances from the donors to the receptors were larger in the 324 and 338K systems than in the 310K system. The rise in temperature increased the entropy, thereby promoting the relative motion of proteins and DNA. Such an increase reduced the stability of hydrogen bonds. Hydrophobic reduction greatly increased the solubility of TtAgo and nucleic acids in solvents, resulting in TtAgo was easier to complete the cutting action of nucleic acid. Overall, the binding of nucleic acid and TtAgo became tighter at higher temperature and TtAgo has high activity at high temperature.

### MM-PBSA calculations

It was well known that the arguably most popular end point method to calculate the free energy of binding between the enzyme and the ligands is the topic of MM/PBSA molecular mechanics [MM] with–Boltzmann [PB] and surface area solvation (Guimarães, 2011). The method has been used in a range of settings, including protein design by free energy calculations. The accuracy is better than for docking and scoring methods (Rifai et al., 2018), comparable to other end point methods, such as LIE methods. Furthermore, it is very fast and thence it was chosen to used for calculated the free energy of binding. The binding free energy of the TtAgo–nucleic acid complex was evaluated using MM–GBSA to confirm the results. The non-bonded van der Waals (ΔE_vdW_), non-bonded electrostatic (ΔE_elec_) interactions, and binding free energy (ΔG_bin_) of the TtAgo–nucleic complex in the three temperature systems are shown in Table 3. The binding free energies were driven primarily by electrostatic interactions and polar solvation, whereas the vdW interaction, polar solvation, and entropy basically remained the same. No significance was found in the variations in the binding affinity between nucleic acid and TtAgo in the different temperature systems. Table 3 shows that the binding free energy of the TtAgo–nucleic complex at 310K (−419.3667 kcal/mol) was higher than those at 324K (−461.1173 kcal/mol) and 338K (−434.8954 kcal/mol). (Seen from Tables S5, S6, it listed the other two repeated simulations results for MM-GBSA Calculations. The results were similar to the results listed in Table 3). These data indicated that the interaction between TtAgo and nucleic acid was more stable at 324 and 338K than at 310K.

**Table 3:** MM-PBSA results (kcal/mol).

	**310K**	**324K**	**338K**
ΔE_ele_	10701.2537	−10983.2665	−11092.4826
ΔE_vdw_	−386.4345	−421.4928	−391.6153
ΔG_np_	−47.0378	−50.5436	−48.4625
ΔG_pb_	10715.3674	10994.1946	11105.0534
ΔE_ele+_ΔE_vdw_	−11087.6982	−11404.7693	−11484.0979
TΔS	10668.3248	10943.6410	11056.5809
ΔG_bind_	−419.3667	−461.1173	−434.8954

**Figure 9:**
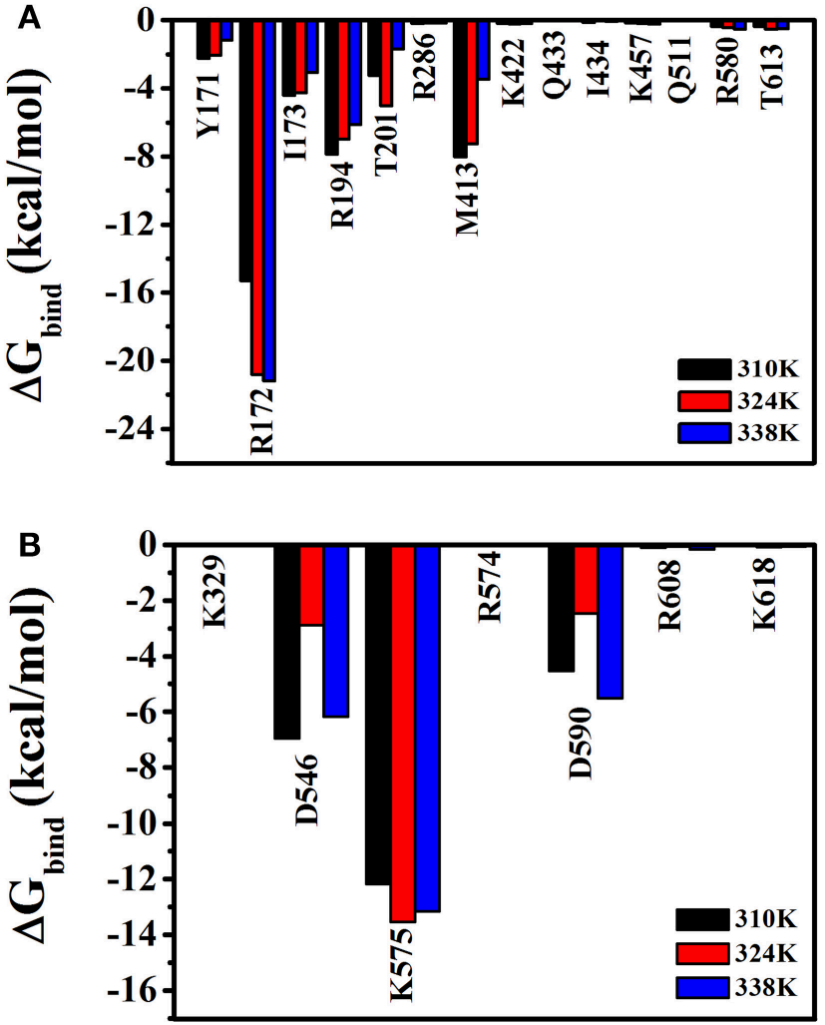
Free energy decomposition analysis at three different temperatures (310 K, 324 K, and 338 K) for TtAgo–nucleic complex. **(A)** The total energy for residues which involved in the interaction with guide DNA. **(B)** The total energy for residues which involved in the interaction with target DNA. The residues that contributed significantly were highlighted.

To analyze the effects of individual amino acids on TtAgo and DNA binding, the per-residue free energy decomposition was performed to characterize and identify the crucial residues that mainly contributed to the binding free energy (Figure 9). Six amino acid residues, namely, Tyr171, Arg172, Ile173, Arg174, Thr174, and Met413 had relatively low energies in the three temperature systems compared with other residues and might play a pivotal role in combining with guide DNA (Figure 9A). It can be seen that Asp546, Lys575, and Asp590 had lower energy which instructed the important role in the process of the combination of target DNA.

## Conclusion

TtAgo is active at high temperatures. The mechanism by which the temperature affects the activity of TtAgo remains poorly understood. In the present study, MD simulations revealed that the disappearance of β-sheet from Glu507 to Gln509 at 310K could cause Glu512 to move far from the catalytic pocket, thereby reducing the activity of TtAgo. The binding channel of the nucleic acid widened at 324 and 338K. Consequently, the binding of nucleic acids and proteins occurred more easily at 324 and 338 K than at 310 K. Binding free energy (MM-PBSA) calculations and hydrogen bond occupancy showed that the binding between TtAgo and nucleic acid was more stable at 324and 338K than at 310 K. These results suggested that the temperature influenced the activity of TtAgo by regulating the binding channel width of TtAgo and the distance from Glu512 to the active center. The activity of TtAgo was higher at 324 and 338K than that at 310K. Our results would be useful as a basis for further studies on the influence of temperature on TtAgo and design of CRISPR methods.

## Author contributions

YL wrote and revised this paper. ZY prepared the tables. JZ made the Supplementary Materials. SW provided some revision advice. DX and WH provided the ideas and modified the papers.

### Conflict of interest statement

The authors declare that the research was conducted in the absence of any commercial or financial relationships that could be construed as a potential conflict of interest.
