# Supplementary figures and images for: Why Is a High Temperature Needed by Thermus thermophilus Argonaute During mRNA Silencing: A Theoretical Study
Source: Front Chem. 2018 Jun 14;6:223. doi: 10.3389/fchem.2018.00223 (PMC6016274; doi:10.3389/fchem.2018.00223)

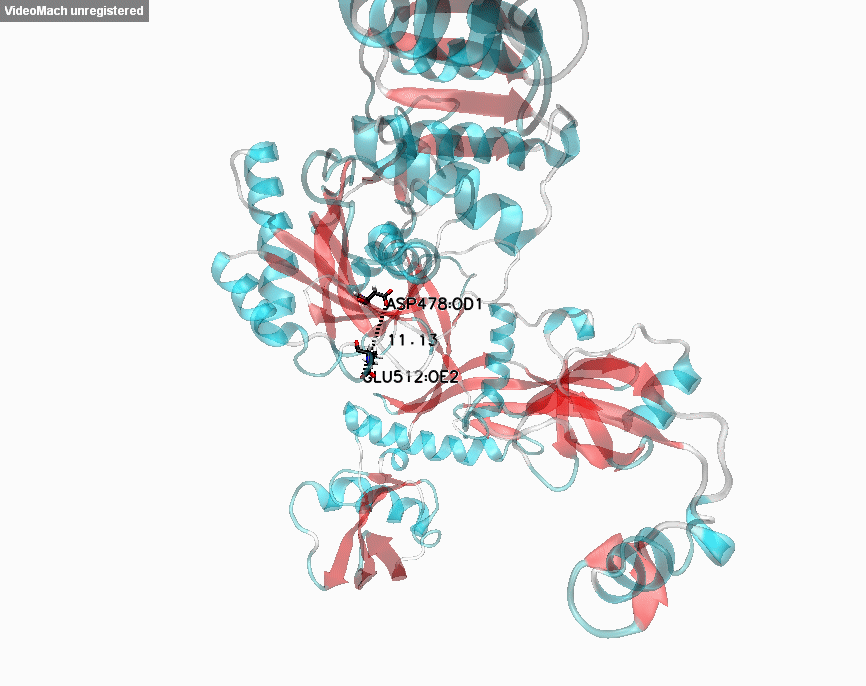

Supplement: Supplementary file 1 [file Data_Sheet_1.ZIP › Visual animation for the distance between Glu512 to Asp478 at 310K.gif]

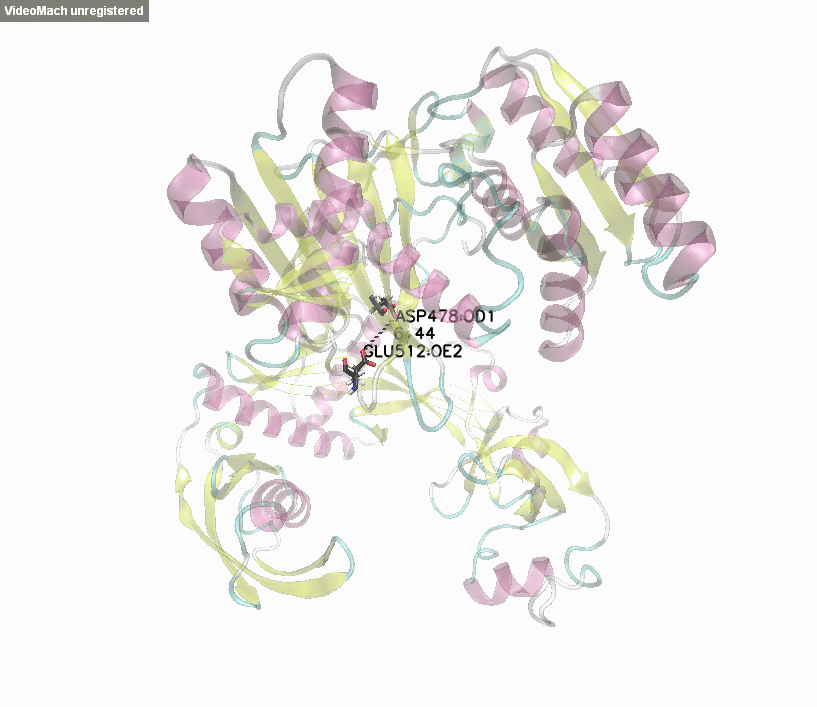

Supplement: Supplementary file 1 [file Data_Sheet_1.ZIP › Visual animation for the distance between Glu512 to Asp478 at 324K.gif]

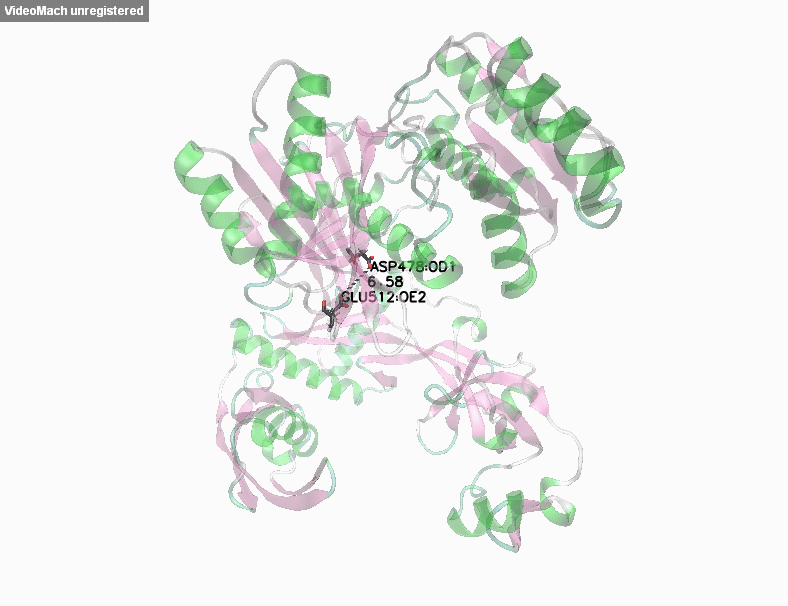

Supplement: Supplementary file 1 [file Data_Sheet_1.ZIP › Visual animation for the distance between Glu512 to Asp478 at 338K.gif]

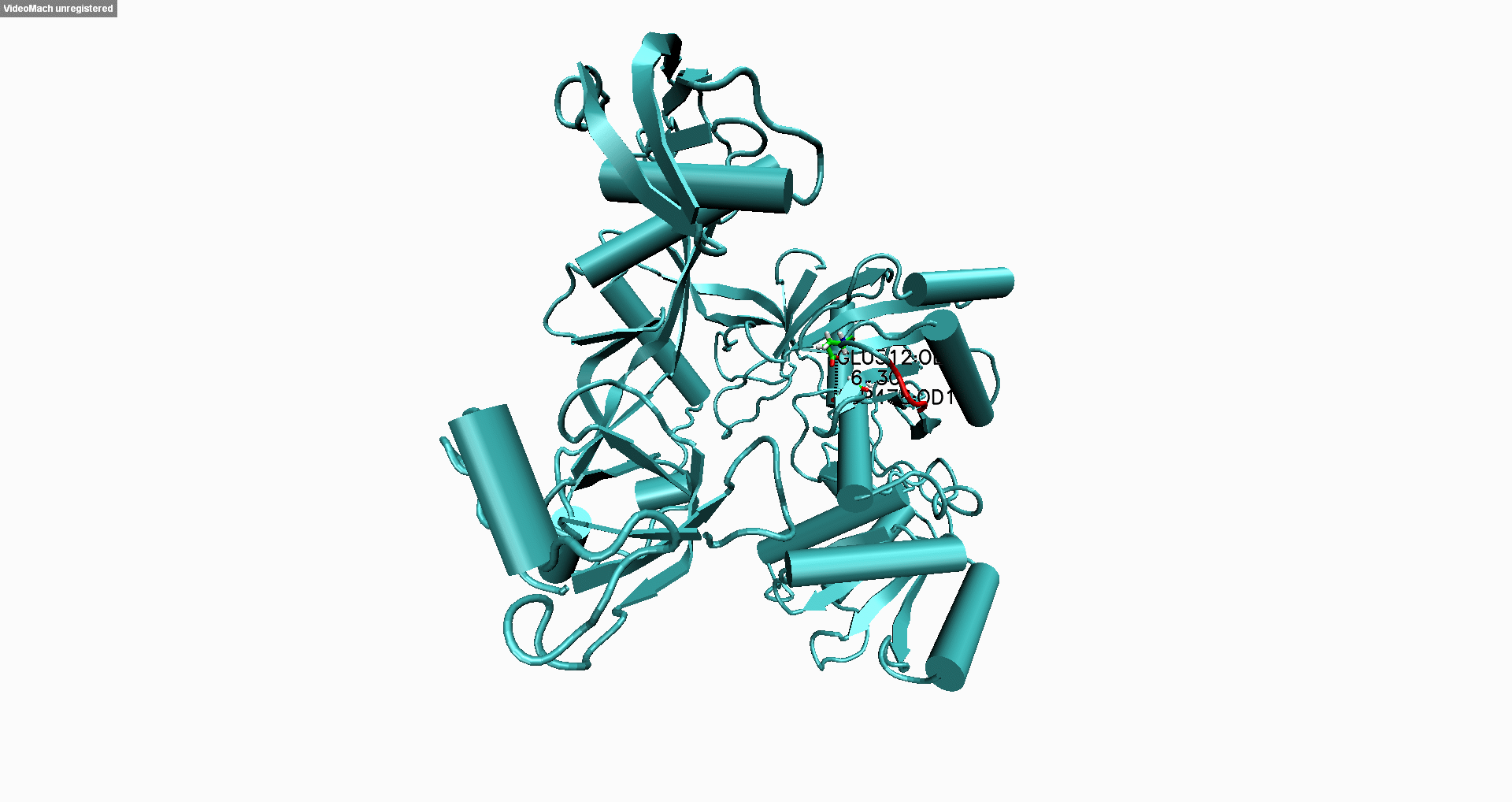

Supplement: Supplementary file 2 [file Data_Sheet_2.ZIP › Visual animation for the distance between Glu512 to Asp478 at 310K.gif]

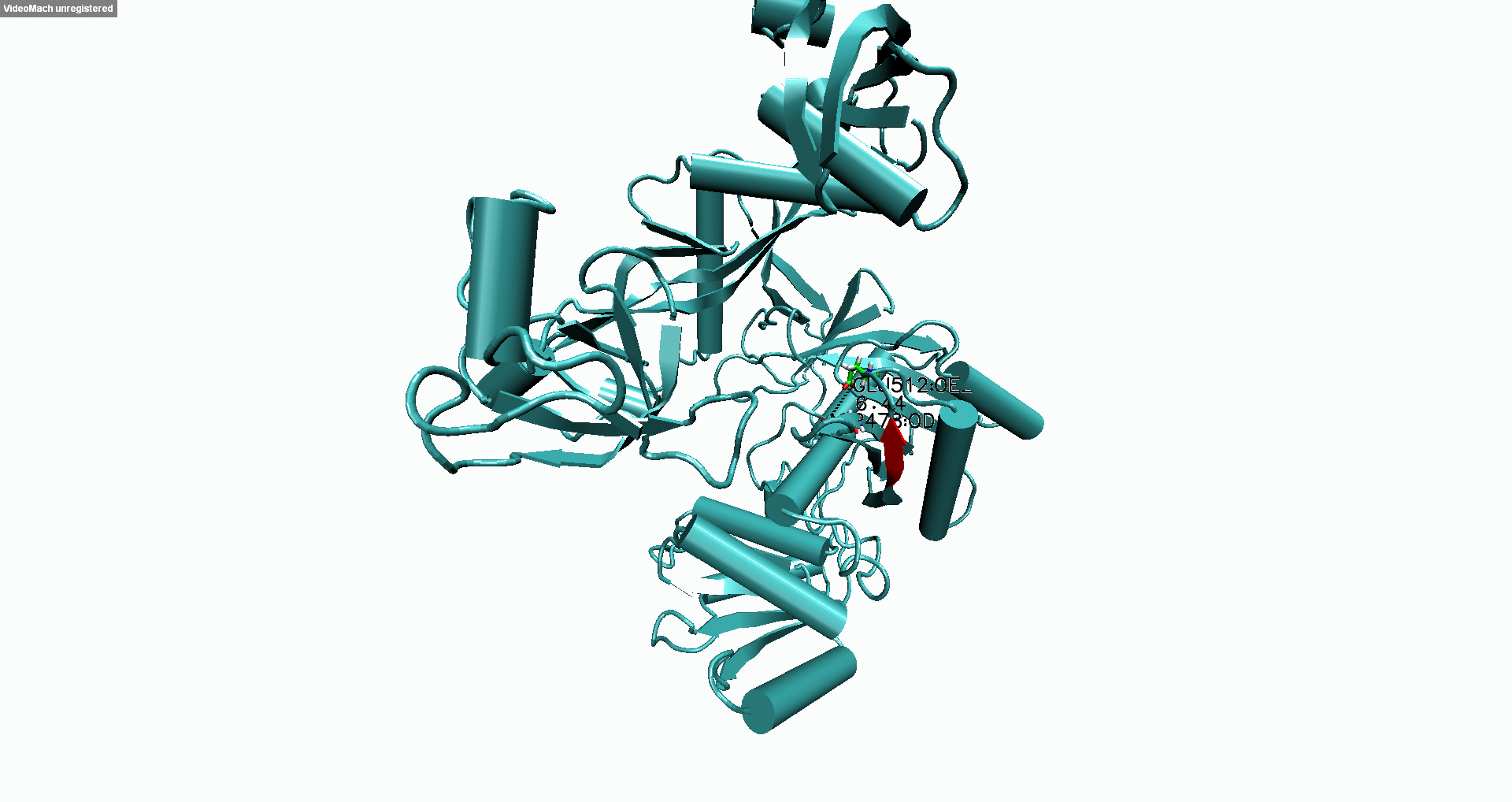

Supplement: Supplementary file 3 [file Data_Sheet_3.ZIP › Visual animation for the distance between Glu512 to Asp478 at 324K.gif]

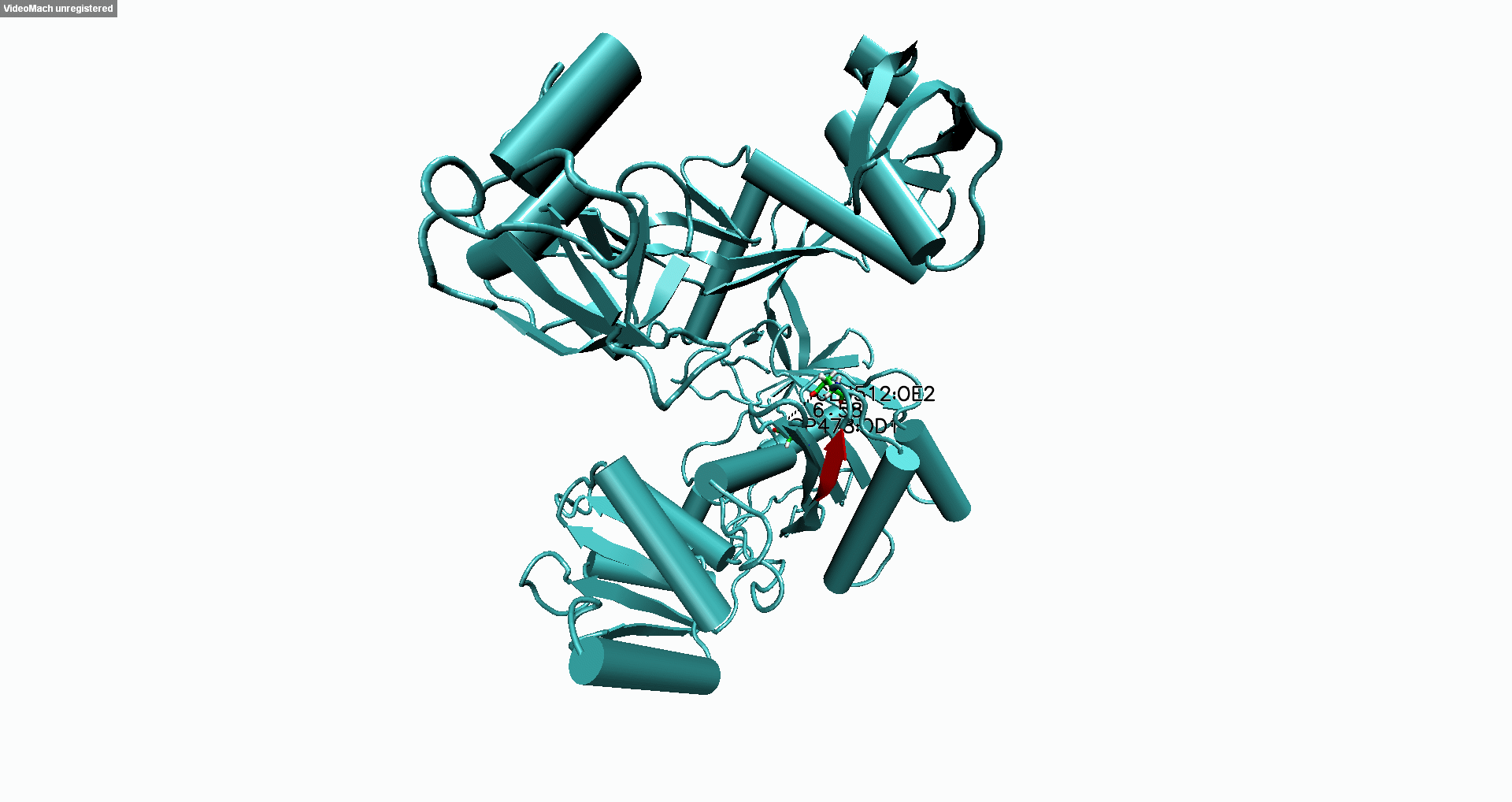

Supplement: Supplementary file 4 [file Data_Sheet_4.ZIP › Visual animation for the distance between Glu512 to Asp478 at 338K.gif]
