# Supplementary material for: Why Is a High Temperature Needed by Thermus thermophilus Argonaute During mRNA Silencing: A Theoretical Study
Source: Front Chem. 2018 Jun 14;6:223. doi: 10.3389/fchem.2018.00223 (PMC6016274; doi:10.3389/fchem.2018.00223)
Supplement: Supplementary file 5 [file Image_1.PDF]

Figure S1 Structural insights from studies of TtAgo ternary complexes with 5′-phosphorylated 16-mer guide DNA and added 15-mer target DNA in the presenceof Mg^{2+} containing solution. (A) Intermolecular contacts in the TtAgo ternary complex with 16-mer target DNA. The interactions highlighted by a yellow background are additional contacts observed beyond those observed in the ternary complex with cleavage-incompatible 15-mer target DNA. (B-C)the nucleic-acid binding channel of TtAgo (structure PDB 4NCB). The seed region (definition in the main text) is enlarged for better visualization in the inset figure. Theguide DNA is in licorice representation while TtAgo atoms in contact with the DNA are plotted as surfaces. Atoms with negative and positive charges are in red and blue, respectively. (D) Structureof the catalytic pocket in the inactive ternary complex with Glu512 positioned outside and far from the catalytic pocket. (E) Structure of the catalytic pocket in the active ternary complex with Glu512 positioned outside and far from the catalytic pocket.

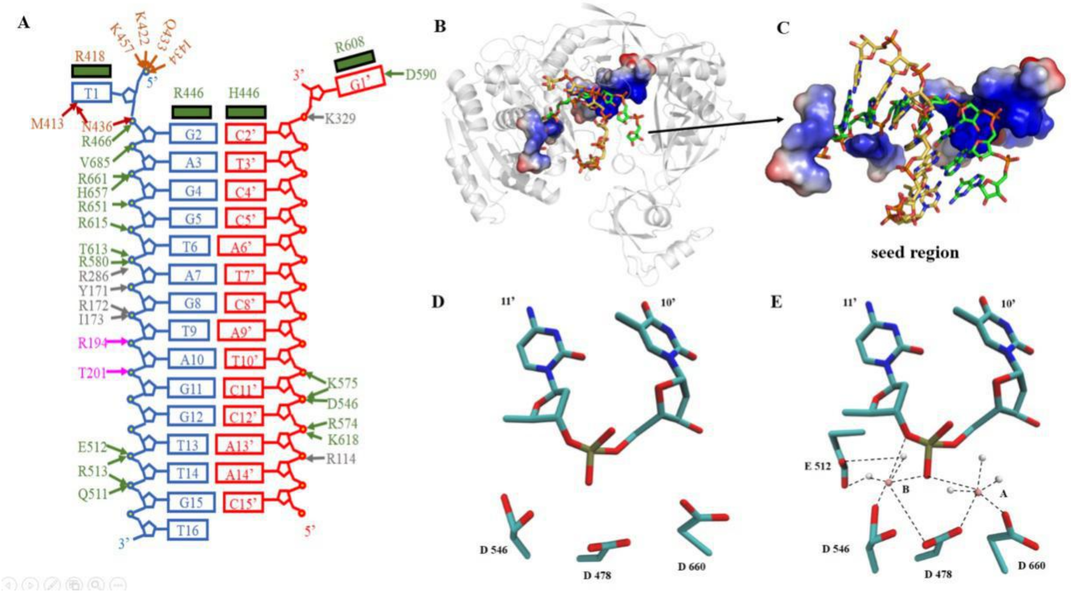

Figure S2 RMSD values of backbone atoms of the whole TtAgo versus simulation time in different temperature systems for three repeat simulations (A-C).

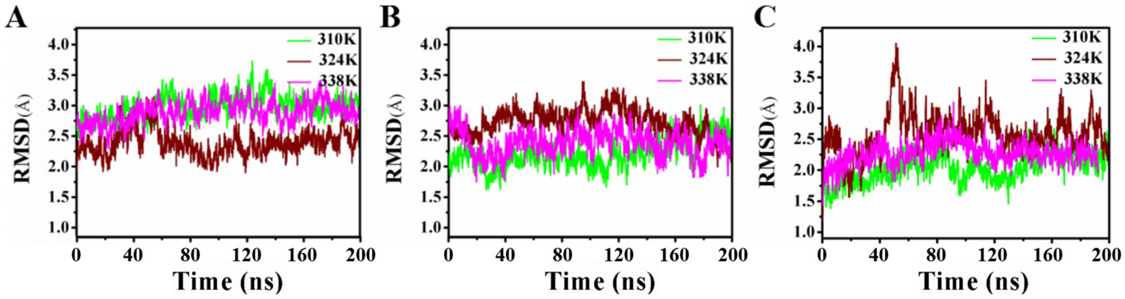

Figure S3 MD trajectories shown as a function of time at different temperature for Total Energy about three systems.

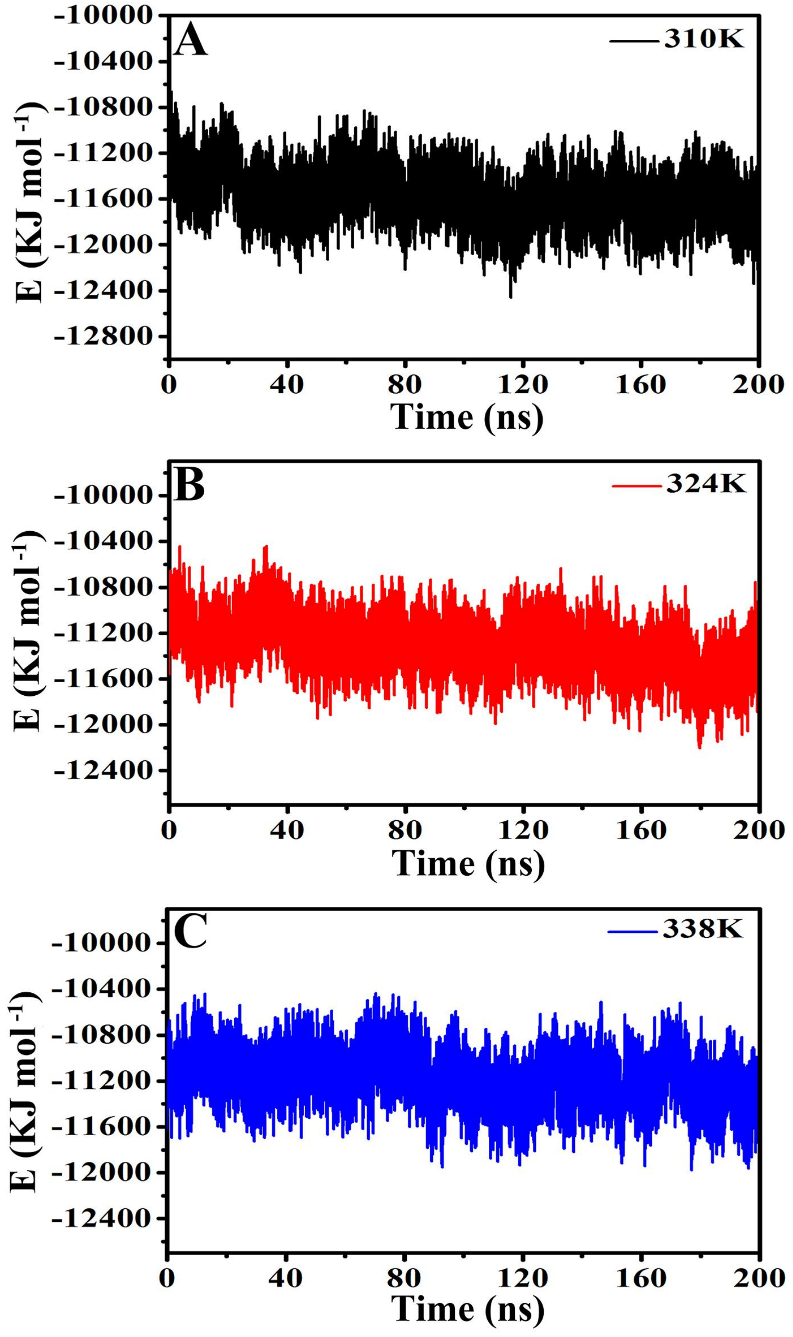

Figure S4 Ramachandran Plot of structures at different time in 310K system. (A-D) were the results of 80.3 ns, 118.8 ns, 145.8 ns and 174.8 ns simulation respectively.

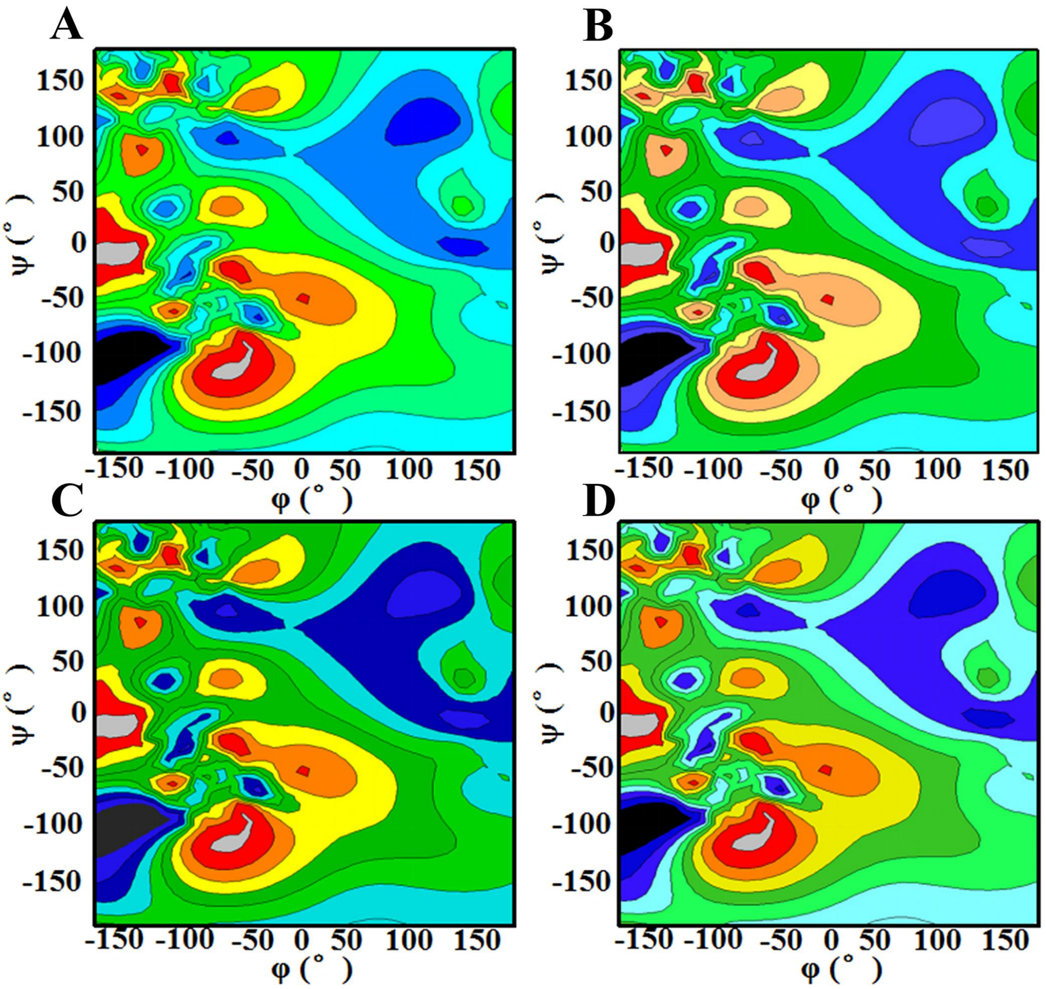

Figure S5 Ramachandran Plot of structures at different time in 324K system. (A-D) were the results of 114.3 ns, 121.6 ns, 162.7 ns and 179.6 ns simulation respectively.

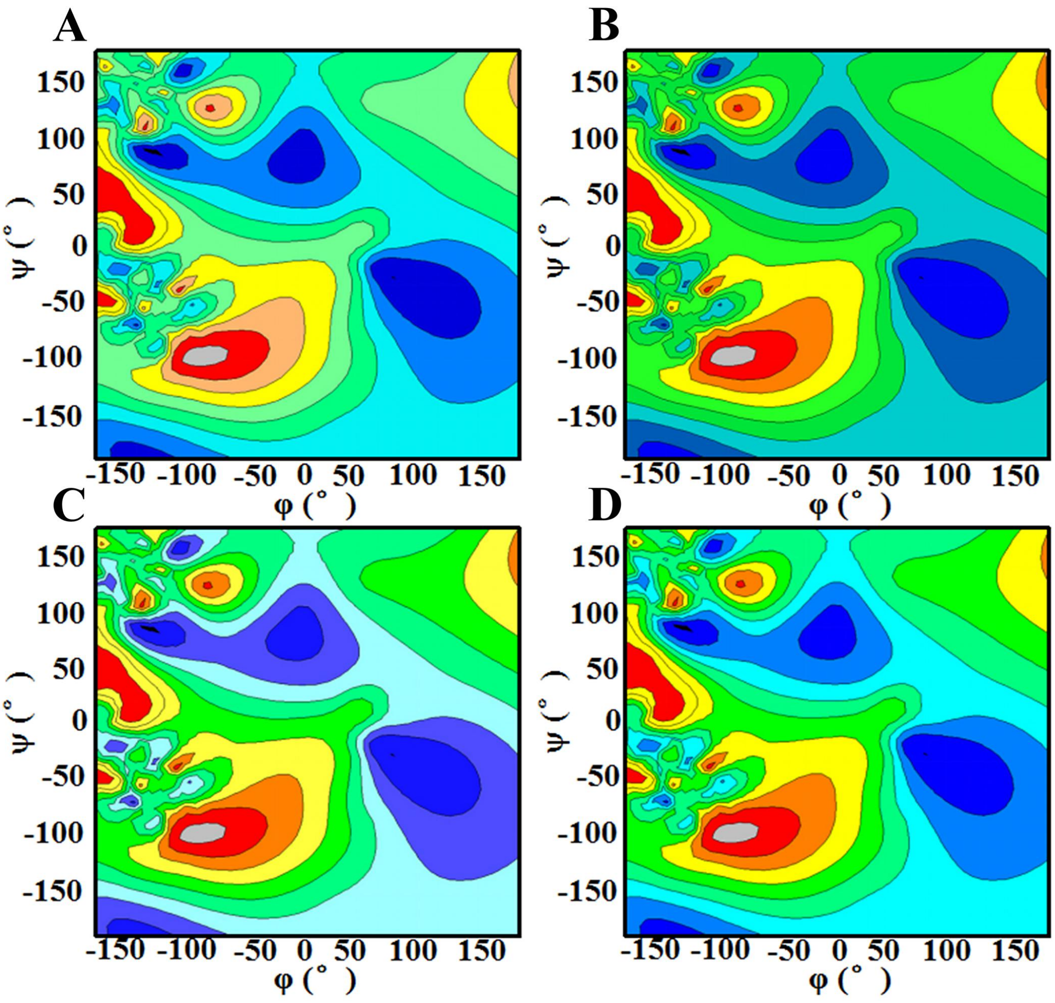

Figure S6 Ramachandran Plot of structures at different time in 338K system. (A-D) were the results of 85.3 ns, 118.5 ns, 163.8 ns and 174.6 ns simulation respectively.

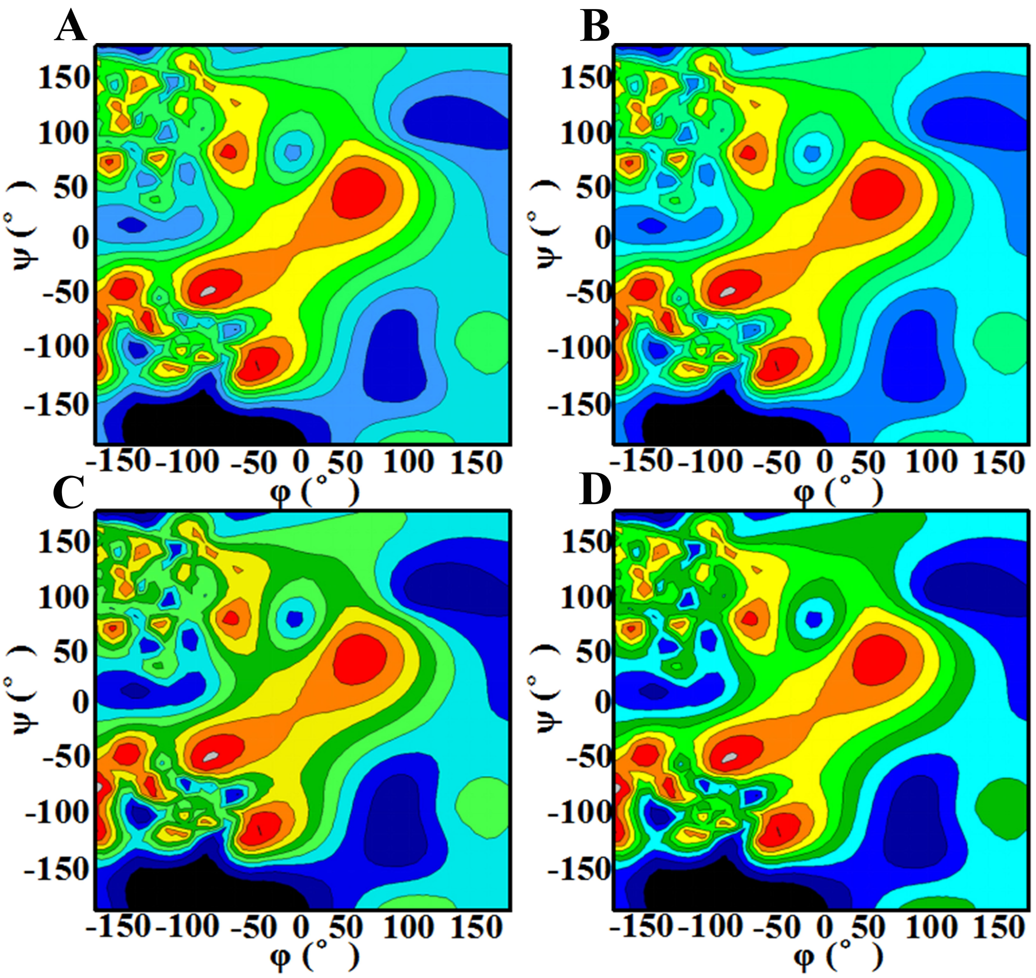

Figure S7 MD trajectories shown as a function of time at different temperature for B-Factor about six domains. The values of B-Factor were calculated according to the formula: $B_A=(8/3)\times\pi^2\times RMSF_A^2$. (A) The B-Factor values of backbone atoms about N terminal. (B) The B-Factor values of backbone atoms about Linker 1. (C) The B-Factor values of backbone atoms about PAZ. (D) The B-Factor values of backbone atoms about Linker 2. (E) The B-Factor values of backbone atoms about MID. (F) The B-Factor values of backbone atoms about PIWI.

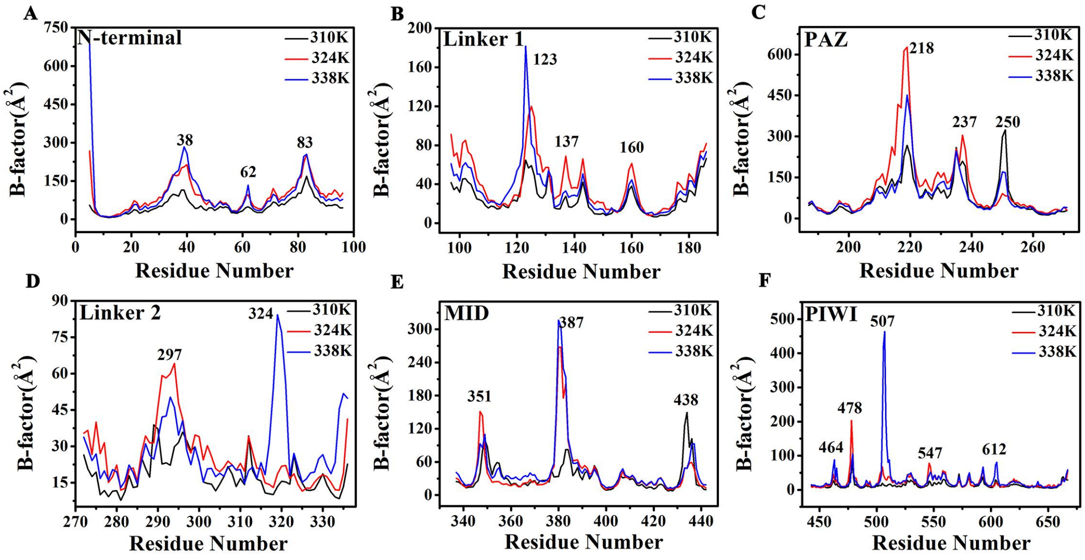

Figure S8 MD trajectories shown as a function of time at different temperature for Radius of gyration (Rg) about six domains. (A) The Rg values of backbone atoms about N terminal. (B) The Rg values of backbone atoms about Linker 1. (C) The Rg values of backbone atoms about PAZ. (D) The Rg values of backbone atoms about Linker 2. (E) The Rg values of backbone atoms about MID. (F) The Rg values of backbone atoms about PIWI.

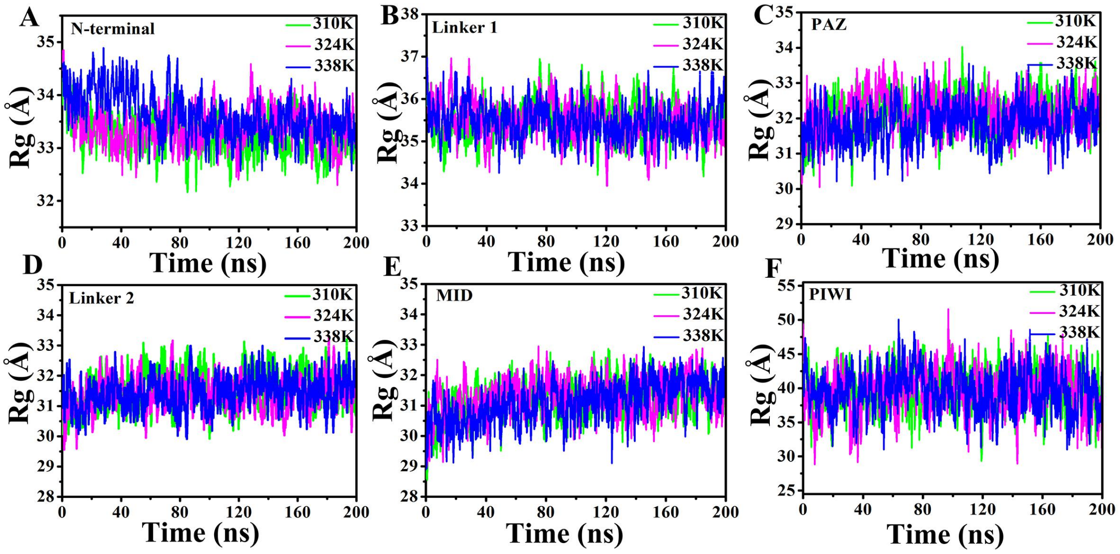

Figure S9 The solvent-accessible surface area (SASA) during 200 ns MD about different TtAgo domain. (A) The SASA values of N terminal calculated in three temperature systems for 200 ns MD trajectories. (B) The SASA values of Linker 1 calculated in three temperature systems for 200 ns MD trajectories. (C) The SASA values of PAZ calculated in three temperature systems for 200 ns MD trajectories. (D) The SASA values of Linker 2 calculated in three temperature systems for 200 ns MD trajectories. (E) The SASA values of MID calculated in three temperature systems for 200 ns MD trajectories. (F) The SASA values of PIWI calculated in three temperature systems for 200 ns MD trajectories.

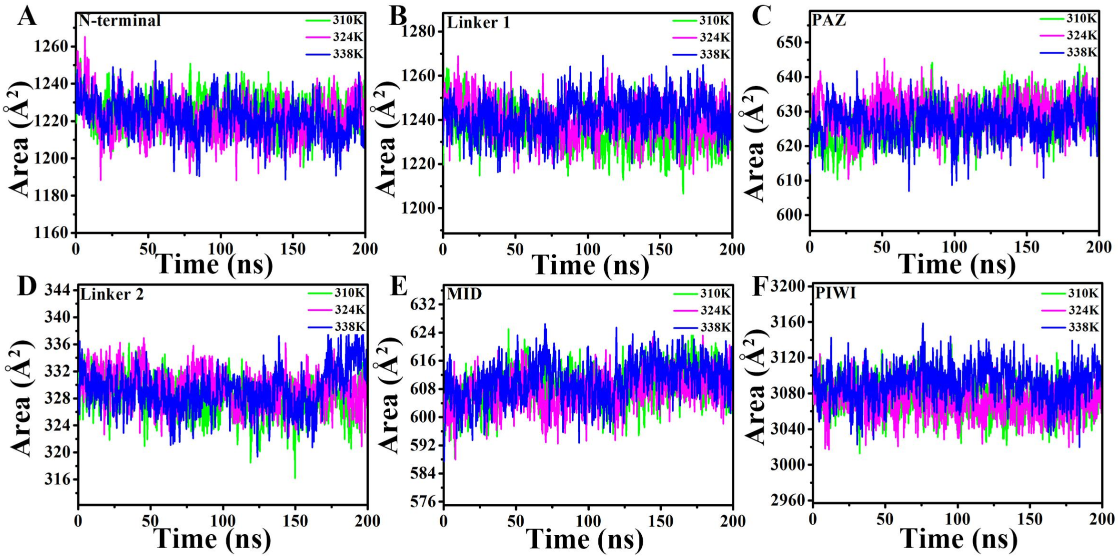

Figure S10 Analysis on residues K575 and D590 for SASA and interaction with nucleic acid at different temperature. (A) SASA for residues K575 and D590 at 310K (black) ,324K (red), and 338K (blue). (B) Interaction between D590 (highlighted by green sticks) and G1′ of target DNA (highlighted by red sticks). The hydrogen bond is represented by the dotted line. (C) Interaction between K575(green sticks) and the C11′ and C12′ of target DNA(highlighted by red sticks). The hydrogen bond is represented by the dotted line.

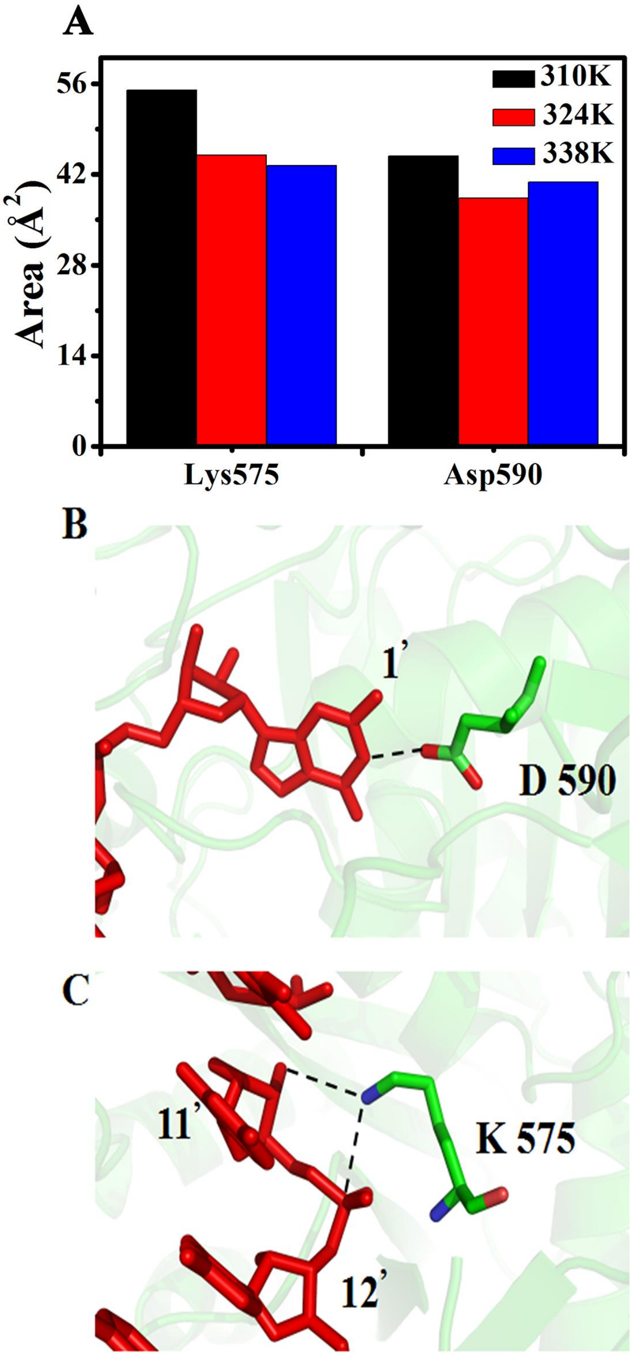

Figure S11 Distance between Glu512 and active pocket in 310K, 324K, and 338K systems for other two repeated simulation. (A-B) Time evolution of distance from Glu512 OE2 to Asp478 OD1 or Asp546 OD1 for the second repeated simulation at three different temperature. (C-D) Time evolution of distance from Glu512 OE2 to Asp478 OD1 or Asp546 OD1 for the third time repeated simulation at three different temperature.

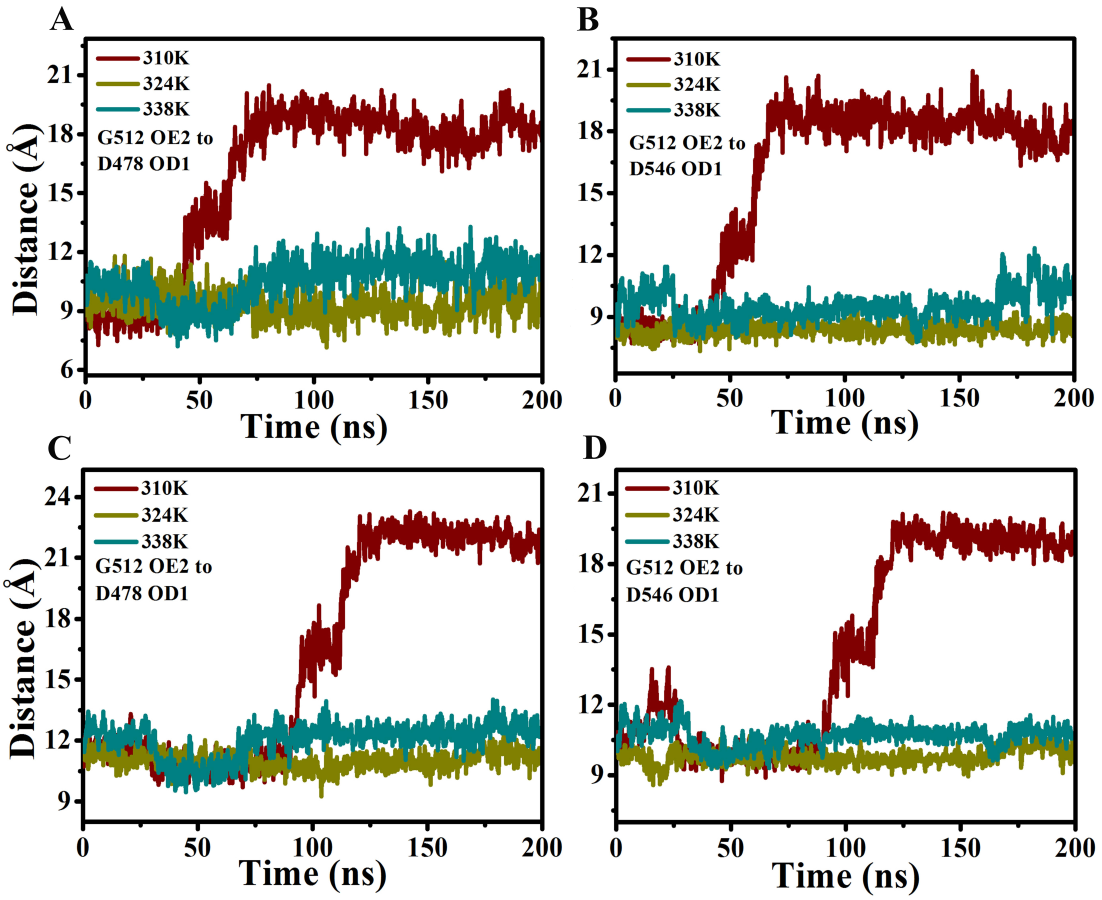

Figure S12 Dynamic changes of the secondary structure profile for TtAgo structure in 310K, 324K and338K systems throughout the simulation. DSSP changes for 310K system (A), 324K system (B) and 338K system (C) during 0-200 ns MD. The color bar represents different secondary structures as follow: 310 - helix (G), α - helix (H), π - helix (I), β - Bridge (B), beta-strand (E), turn (T), coil (C).

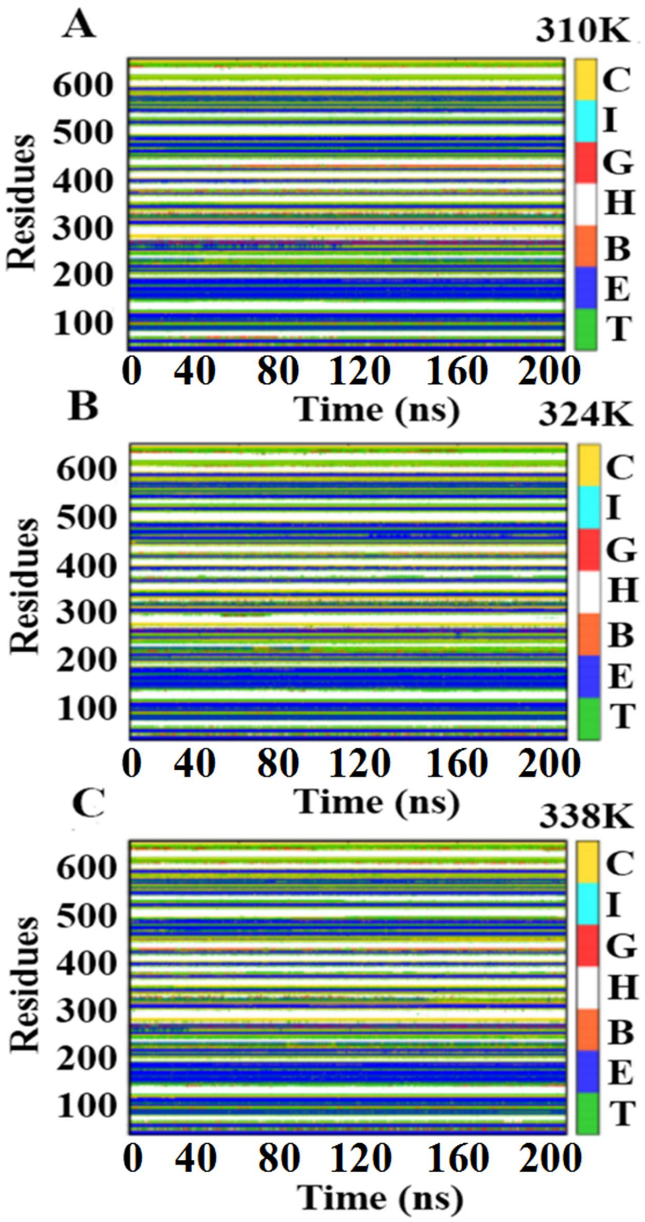

Table S1 Probability of generating β-bridge in E512 loop and D546 loop for the second repeated simulation

System	E512 loop	B-brige occupancy
310K	E507 to Q509	1.1%
324K	E507 to Q509	45.7%
338K	E507 to Q509	38.9%

Table S2 Probability of generating β-bridge in E512 loop and D546 loop for the third time repeated simulation

System	E512 loop	B-brige occupancy
310K	E507 to Q509	1.7%
324K	E507 to Q509	46.3%
338K	E507 to Q509	44.1%

Table S3 Hydrogen bond occupancy between Target and TtAgo during 200 ns MD simulations

Hydrogen Bonds Occupancies		310K(%)	324K(%)	338K(%)
Donor	Accepter			
ARG59:NH2	Target DNA DC5:O2'	80.09	80.09	
ARG59:NE	Target DNA DC5:O2'	71.24		
Target DNA	DC8:O2'ARG548:NH1	98.34	91.54	66.79
SER576:CB	Target DNA DC9:O1P	91.34	51.92	91.83
SER576:OG	Target DNA DC9:O1P	99.72	99.90	97.87
SER576:N	Target DNA DC9:O1P	94.19	80.19	98.05
LYS575:N	Target DNA DC9:O2P	100.00	100.00	98.05
LYS575:NZ	Target DNA DC9:O2P	99.26		60.48
LYS575:NZ	Target DNA DC9:O5'	84.24		50.06
GLY547:CA	Target DNA DC9:O5'	76.68		
LYS575:CE	Target DNADT10:O1P	93.18	80.19	63.59
LYS575:NZ	Target DNADT10:O1P	98.53		
GLY481:N	Target DNA DT10:O3'	61.75		55.67
LYS664:NZ	Target DNADA11:O1P	99.72		98.31
LYS664:CG	Target DNA DA11:O1P	51.80		
GLY480:CA	Target DNADA11:O2P	50.41		66.52
ARG668:NH1	Target DNADC12:O2P	85.62		78.86
PHE647:CD2	Target DNADC18:O3'	55.39	50.10	
HSE607:O	Target DNADG19:N1	88.57	51.73	58.70
Target DNA	PHE647:O	89.03		
Target DNA	ASP590:OD2	95.30	88.17	84.99
Target DNA	ASP590:OD1	89.68		
Target DNADG19:N1	ASP590:OD1	75.48		
Target	VAL606:CG1	51.24	62.69	

Table S4 Hydrogen bond occupancy between Target and TtAgo during 200 ns MD simulations

Hydrogen Bonds Occupancies		310K(%)	324K(%)	338K(%)
Donor	Accepter			
THR613:N	Guide DNADT6:O3'	50.05		
ARG286:NH2	Guide DNA DA7:O2P	97.60		
ARG580:NH1	Guide DNA DA7:O1P	98.34	98.94	92.45
ARG446:NH1	Guide DNA DG2:O1P	89.86		85.08
ARG615:N	Guide DNA DT6:O2P	59.54	97.79	
ASN449:ND2	Guide DNA DA3:O4'	95.58	96.35	79.40
TYR171:N	Guide DNA DG8:O2P	86.91	91.54	98.76
ARG615:NE	Guide DNADT6:O1P	78.25		
ASN436:N	Guide DNA DG2:O1P	100.00	99.13	69.80
ARG615:NH2	Guide DNA DT6:O1P	98.62		57.37
GLN433:NE2	Guide DNA DG2:O3'	50.51		
ARG651:N	Guide DNA DG5:O2P	99.26	99.23	96.18
ARG651:CB	Guide DNA DG5:O2P	56.96	55.77	
PRO650:CA	Guide DNA DG4:O3'	52.63		
ILE173:N	Guide DNA DT9:O2P	99.82	94.42	95.47
HSE657:NE2	Guide DNA DG4:O2P	99.91		93.96
TYR642:OH	Guide DNA DG4:O2P	99.82		73.00
GLN433:NE2	Guide DNA DA3:O2P	62.58		
LYS457:NZ	Guide DNA DA3: O2P	98.25		
MET413:N	Guide DNA DT1: O2	85.62	56.06	54.80
Guide DNA DT6: C3'	THR613:O	90.32	74.04	
Guide DNA DT1:N3	MET413:O	100.00	97.98	72.20
THR613:N	Guide DNA DA7: O2P	95.21	65.10	73.36
ARG194:NH2	Guide DNA DA10 :O2P	58.80		
ARG548:NE	Guide DNA DT14: O4'	96.50		
GLY511:CA	Guide DNADT14:O2P	51.43		

Guide DNA DA3:C5'	ALA644: O	55.30
THR613:CB	Guide DNA DA7:O2P	57.60
GLY453: CA	Guide DNA DA3: O2P	52.17
TYR642: CE2	Guide DNA DG4:O2P	57.97

Table S5 MM-PBSA results (kcal/mol) for the second repeated simulation

	310K	324K	338K
ΔE_{ele}	-10769.6358	-10978.4927	-10935.6952
ΔE_{vdw}	-393.2514	-404.2487	-412.7463
ΔG_{np}	-50.3026	-56.9854	-55.7542
ΔG_{pb}	10865.6743	11044.9635	11014.8756
$\Delta E_{ele} + \Delta E_{vdw}$	-11162.8872	-11382.7414	-11366.4415
$T\Delta S$	10815.3717	10987.9781	10959.1214
ΔG_{bind}	-347.5155	-394.7633	-409.3201

Table S6 MM-PBSA results (kcal/mol) for the the third time repeated simulation

	310K	324K	338K
ΔE_{ele}	-10637.3678	-10894.4369	-10938.8764
ΔE_{vdw}	-375.6848	-395.5463	-383.9837
ΔG_{np}	-43.0543	-45.3549	-47.8356
ΔG_{pb}	10657.7864	10859.4629	10948.9463
$\Delta E_{ele} + \Delta E_{vdw}$	-11013.0526	-11298.9832	-11322.8601
$T\Delta S$	10614.7321	10814.1080	10901.1107
ΔG_{bind}	-398.3205	-484.8752	-421.7494
